# Hydrogen Bond Interaction Networks in the Mixed Pentamers of Hydrogen Sulfide and Water

**DOI:** 10.1021/jacs.4c18276

**Published:** 2025-05-26

**Authors:** Pablo Pinacho, Cristóbal Pérez, Marcel Stahn, Rizalina T. Saragi, Andreas Hansen, Stefan Grimme, Alberto Lesarri, Melanie Schnell

**Affiliations:** † 28332 Deutsches Elektronen-Synchrotron DESY, Notkestr. 85, 22607 Hamburg, Germany; ‡ Departamento de Química Física y Química Inorgánica, Facultad de Ciencias − I.U. CINQUIMA, 16782 Universidad de Valladolid, Paso Belén 7, 47011 Valladolid, Spain; § Mulliken Center for Theoretical Chemistry, Institut für Physikalische und Theoretische Chemie, 9374 Rheinische Friedrich-Wilhelms-Universität Bonn, Beringstraße 4, 53115 Bonn, Germany; ∥ Chemical Sciences and Engineering Division, 1291 Argonne National Laboratory, Lemont, Illinois 60439, United States; ⊥ Institut für Physikalische Chemie, Christian-Albrechts-Universität zu Kiel, Max-Eyth-Str. 1, 24118 Kiel, Germany

## Abstract

The observation of gas-phase water clusters has been instrumental in understanding water aggregation and cooperativity, paving the way for solvation models in the bulk. However, the characterization of hydrogen sulfide self-aggregation is still largely unexplored. Here, we investigate two mixed pentamers of hydrogen sulfide and water to examine the influence of the weaker, dispersion-based and less directional interactions caused by hydrogen sulfide. Unprecedented structural resolution was obtained by combination of jet-cooled broadband rotational spectroscopy and quantum-chemical calculations. Specifically, we compare the 4:1 and 1:4 hydrogen sulfide - water pentamers, offering comparison with the prototype homoclusters. Important structural differences are revealed in the hydrogen sulfide clusters, which reorganize to compensate for the weaker sulfur-centered hydrogen bonds. The noncovalent interactions in the pentamers were rationalized using density functional theory and reduced electronic density calculations. Moreover, a comprehensive many-body decomposition energy analysis revealed significant variations in molecule two- and three-body contributions to the total interaction energy based on the relative proportions of H_2_O and H_2_S. These findings offer new insights into the distinct cooperative forces in water and hydrogen sulfide clusters. The results will improve our understanding and modeling of sulfur-centered hydrogen bonds, which may be useful across various research fields, including protein folding, molecular aggregation, materials science, and computational benchmarking.

## Graphical abstract

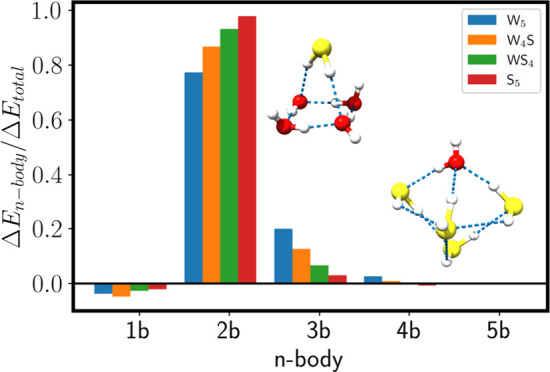

## Introduction

The striking difference between liquid water and gaseous hydrogen sulfide is a textbook example of the relevance of hydrogen bonding. While oxygen and sulfur are isovalent chalcogens, their electronic properties translate into different electronegativities and polarizabilities, resulting in distinctive intermolecular interactions and macroscopic properties of the two dihydrides. Extensive endeavors have been dedicated to study water from a molecular point of view,^1,2^ but still no model offers a satisfactory connection between its microscopic molecular properties and the bulk.^3^ The most useful molecular strategy is disaggregation by analyzing small size-controlled water clusters in the gas phase at low temperatures, typically in a jet expansion. The combination of high-resolution spectroscopy experiments and quantum-chemical calculations has now progressed from the water dimer^4–7^ to the decamers,^8–15^ offering detailed pictures on hydrogen bond aggregation patterns,^8–11,13,14^ internal dynamics,^10,12^ and cooperativity^9,15^ that may guide the construction of theoretical models for larger adducts. Among gas-phase techniques, jet-cooled microwave spectroscopy provides detailed structural insights of molecules and complexes, enabling a direct characterization of intermolecular interactions through the moments of inertia, free of solvation or crystal packing effects.^16–18^

Conversely, information on gas-phase hydrogen sulfide aggregates is practically absent, despite sulfur’s prevalence and its important chemical role. Hydrogen bonds involving sulfur play an important role in proteins, molecular assemblies and functional materials,^19–21^ and their relative strength compared to the oxygen counterparts has been debated.^22–24^ Most of the existing gas-phase information concerns the thiol group acting as proton acceptor, as in the O–H···S,^25–28^ N–H···S,^29–32^ and C–H···S^33,34^ hydrogen bonds. However, the observations of thiols as proton donor (S–H···O,^35–37^ S–H···N,^38^ S–H···π,^39–41^ etc.) or the elusive S–H···S hydrogen bond^42–48^ are limited to a few cases. The experimental information on hydrogen sulfide aggregates is even more scarce and restricted to the (H_2_S)_2_ dimer,^35,42–44^ the (H_2_S)_2_···(H_2_O) trimer^49^ and several argon complexes (Ar···(H_2_S),^50^ Ar_2_···(H_2_S),^51^ and Ar_3_···(H_2_S)^52^). A vibrational study recently reported the observation of the dimer, trimer, and tetramer in a cold argon matrix,^48^ but its low resolution makes the rotational investigation of larger hydrogen sulfide clusters a fundamental topic for noncovalent interactions.

The water trimer, tetramer and pentamer feature cyclic near-planar ring geometries of the oxygen atoms and, therefore, are nonpolar and not detectable by rotational spectroscopy.^8–15^ However, the potential energy surface (PES) of hydrogen sulfide is more complex. Previous theoretical studies explored the (H_2_S)_*n*_ complexes up to the pentamer,^53–55^ and its mixed clusters with water up to the tetramer,^56^ showing that in the presence of hydrogen sulfide three-dimensional structures are preferred over the planar rings in the tetramer and pentamer. The addition of even just one H_2_S molecule to the water structures induces a transition from two-dimensional to three-dimensional heavy-atom shapes, enabling their analysis by rotational spectroscopy.

We report here on two water-mixed pentamers of hydrogen sulfide, with the objectives of studying the nature of the sulfur-centered intermolecular interactions, the formation of cooperative hydrogen-bonded networks, the cluster-growth mechanisms, and the differences with the water prototypes. Since computational methods perform worse for the heavier chalcogens compared to oxygen, the experimental studies may serve also as benchmarking references for the tasks of conformational search and structural and energetic determination of their shallow PES.

These mixed clusters offer an exceptional opportunity to observe the molecular effects of introducing a competing chalcogen molecule into the homomeric structures, shedding light on the electronic and structural differences. At the same time, the asymmetry introduced by the different chemical species eliminates tunneling effects arising from large-amplitude motions. This fact reduces the complexity of the spectral analysis and makes them suitable for the investigation of their aggregation properties and benchmarking of computational methods. In addition, the mixed dimers are useful gauges of the relative strength of the intermolecular interactions of hydrogen sulfide and water within a single cluster as well as of the occurrence of cooperative effects.

## Results

### Rotational Spectra of the Mixed Pentamers

The microwave spectrum of hydrogen sulfide and water in Figure 1 is very complex and congested (roughly 6200 transitions with signal-to-noise ratio above 3:1 in the 2–12 GHz region), containing rotational signatures from multiple clusters of different size and chemical composition. This included, despite having weak intensities, rotational transitions from water clusters up to the heptamer. Some of the most prominent lines, apart from those from the hydrogen sulfide dimer, were soon attributed to pentameric species, suggesting these species for an initial study. In this report, we present the results concerning the (H_2_O)-(H_2_S)_4_ and (H_2_O)_4_-(H_2_S) mixed pentamers (later abbreviated WS_4_ and W_4_S, respectively). The pure hydrogen sulfide pentamer could not be assigned because of extensive tunnelling effects hindering the spectral analysis.

**Figure 1:**
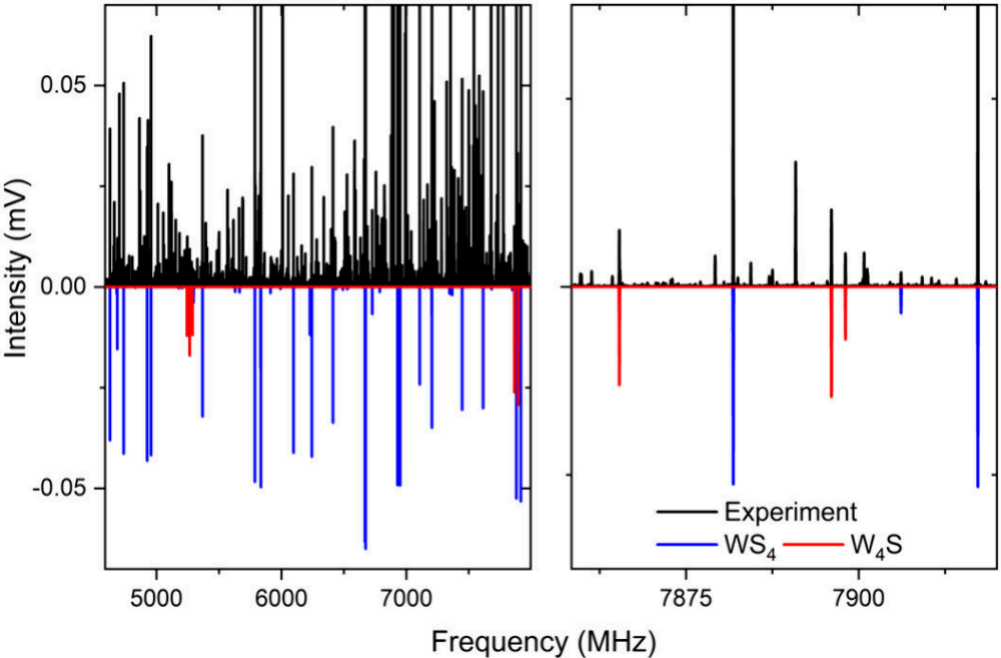
Left panel shows a section of the experimental rotational spectrum of a gas mixture of H_2_S and H_2_O, highlighting the high density of lines. The right panel shows selected rotational transitions for WS_4_ and W_4_S clusters in blue and red, respectively. The colored traces are simulations with a rotational temperature of 2 K based on the fitted rotational parameters in Tables 1 and 2.

Different computational models were used in this study, including B3LYP-D3­(BJ)/def2-TZVP,^57–59^ ωB97X-V/def2-TZVP,^60^ and MP2/aug-cc-pVTZ.^61–63^ A complete description of the computational methodology and theoretical results is given in the Supporting Information. The conformational search focused on the detection of the global minimum and the lowest energy isomers. For both pentamers W_4_S and WS_4_, the computational methods predicted three isomers in a narrow energy range below 3 kJ·mol^–1^ (Tables S1–S2, Figure 2).

**Figure 2:**
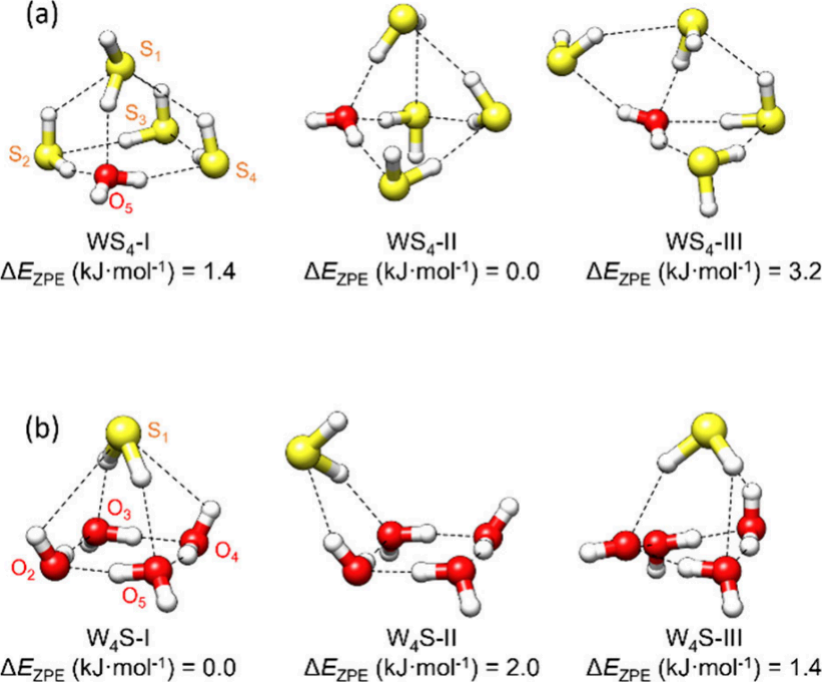
Predicted lowest-energy structures for WS_4_ (a) and W_4_S (b) (ωB97X-V/def2-TZVP), including the relative electronic energy with zero-point energy correction.

The rotational spectrum was first examined to exclude previously identified species. Once the transitions belonging to the hydrogen sulfide dimer^43^ were removed from the spectrum, some intense lines showing characteristic *a-*, *b*-, and *c*-type quartets were first identified. Since the rotational constants critically depend on the cluster size and geometry, the (ground-state) experimental values were compared with the (equilibrium) computational predictions. The experimental transition intensities provided a second discrimination argument (Table 1, Figure S1). Both comparisons showed an excellent agreement with the predictions for the WS_4_–I isomer of the mixed pentamer (H_2_O)_1_-(H_2_S)_4_, confirming the detection of this species. The later observation of the ^34^S isotopologues in natural abundance (4.3%) and the determination of the experimental structure definitively confirmed the assignment.

**Table 1:** Rotational Parameters for (H_2_O)-(H_2_S)_4_, Comparing the Experimental Results with the Three Most Stable Isomers (ωB97X-V/def2-TZVP Level)

	Exp.	WS_4_-I	WS_4_-II	WS_4_-III
*A*/MHz^a^	865.25110(11)^b^	875	824	1077
*B*/MHz	657.994990(95)	684	596	516
*C*/MHz	564.323353(88)	576	585	460
Δ_*J*_/kHz	0.53003(57)	0.33^c^	0.61	0.20
Δ_*JK*_/kHz	–1.3627(11)	–0.64	5.34	1.28
Δ_*K*_/kHz	2.9068(12)	1.49	–3.55	–0.30
δ_*J*_/kHz	0.14284(18)	0.09	–0.01	0.01
δ_*K*_/kHz	–0.2598(20)	–0.15	8.60	1.32
μ_a_/D	**	1.2	1.5	1.0
μ_b_/D	***	3.0	0.8	1.4
μ_c_/D	*	0.3	2.4	0.1
*N*	260	–	–	–
σ/kHz	5.4	–	–	–
Δ*E*_ZPE_/kJ·mol^–1^	–	1.4	0.0	3.2

a Rotational constants (*A*, *B*, *C*), quartic centrifugal distortion constants (Δ_*J*_, Δ_*JK*_, Δ_*K*_, δ_*J*_, and δ_*K*_), electric dipole-moment components (μ_a_, μ_b_, and μ_c_) and experimental intensities (proportional to the number of asterisks), number of fitted transitions (*N*), root-mean square deviation of the fit (σ) and relative electronic energy including the zero-point energy correction (Δ*E*_ZPE_).

b Standard errors in parentheses in units of the last digit.

c The centrifugal distortion constants were computed within the harmonic approximation at the ωB97X-D/def2-TZVP level of theory.

Due to their small energy difference, isomers WS_4_-II and WS_4_-III may be populated in the jet, but they were not observed. No large-amplitude motions were observed in the spectrum of WS_4_-I, so the spectrum of this isomer could be reproduced with a semirigid rotor model.^64^ The fitted rotational parameters (rotational constants and quartic centrifugal distortion constants) are summarized in Table 1.

Further exploration of the spectrum resulted in the fit of a weaker pentamer cluster (in red in Figure 1), producing the rotational parameters given in Table 2. The comparison with the predicted parameters and the observation of only *a*-type transitions (Table 2, Figure S1) confirmed the identification of the second experimental species as the global minimum of the pentamer (H_2_O)_4_-(H_2_S)_1_, denoted isomer W_4_S–I in Figure 2. Some quartic centrifugal distortion constants for this cluster could not be determined because of the reduced number of transitions.

**Table 2:** Rotational Parameters for (H_2_O)_4_-(H_2_S), Comparing the Experimental Results with the Three Most Stable Isomers (ωB97X-V/def2-TZVP Level)

	Exp.	W_4_S–I	W_4_S–II	W_4_S–III
*A*/MHz^a^	1772.37(47)^b^	1823	2571	2042
*B*/MHz	1327.6340(93)	1410	975	1292
*C*/MHz	1305.4979(90)	1352	835	1259
Δ_*J*_/kHz	1.547(61)	1.04^c^	1.05	1.28
Δ_*JK*_/kHz	48.14(29)	4.87	–3.63	–1.21
Δ_*K*_/kHz	[0]	–5.36	10.82	3.00
δ_*J*_/kHz	[0]	0.05	0.03	–0.07
δ_*K*_/kHz	17.8(45)	3.15	0.56	–5.29
μ_a_/D	***	1.1	1.2	2.7
μ_b_/D	–	0.0	1.0	2.6
μ_c_/D	–	0.0	0.6	0.3
N	16	–	–	–
σ/kHz	6.2	–	–	–
Δ*E*_ZPE_/kJ·mol^–1^	–	0.0	2.0	1.4

a Parameter definition as in Table 1.

b Standard errors in parentheses in units of the last digit.

c The centrifugal distortion constants were computed within the harmonic approximation at the ωB97X-D/def2-TZVP level of theory.

A first comparison of the WS_4_ and W_4_S isomers is pertinent here. W_4_S is characterized by a homodromic water hydrogen-bonded network similar to the pure water tetramer and one out-of-plane sulfur atom. In this geometry, inversion of the water orientation simply produces the enantiomeric species. However, for the lowest energy isomer of WS_4_ there are additional degrees of freedom associated to the nonsequential heterodromic hydrogen-bonded network. In consequence, the orientations of the hydrogen atoms generate multiple isomers within the same structural family. However, the slightly different isomers still possess unique rotational constants, dipole-moment components and energies permitting their unequivocal identification (Table S3, Figure S2).

From the three levels of theory employed, the hybrid generalized-gradient-approximation functional ωB97X-V/def2-TZVP gave the best agreement with the experiment, with relative differences in the rotational constants around 4%. The reasons for the better performance compared to higher levels of theory are unclear and could be due to beneficial error cancellation.

### Molecular Structure

The structure of the WS_4_ pentamer could be assessed using the observations of the four ^34^S monosubstituted isotopologues (Table S4, Figures S3–S4). This information enabled the determination of the experimental structure, displayed in Figure 3, through the substitution (*r*_s_) and effective (*r*_0_) methods.^65,66^ For the determination of the *r*_0_ structure, the monomers were assumed to be unperturbed upon complexation and the distances and angles between the heavy atoms in the cluster were floated, while the rest of the parameters were fixed to their theoretical values at the ωB97X-V/def2-TZVP level. In the *r*_s_ structure, the isotopic differences of the moments of inertia are used in the Kraitchman equations to obtain near-equilibrium coordinates. The results of structure determination are reported in detail in the SI (Tables S5–S8).

**Figure 3:**
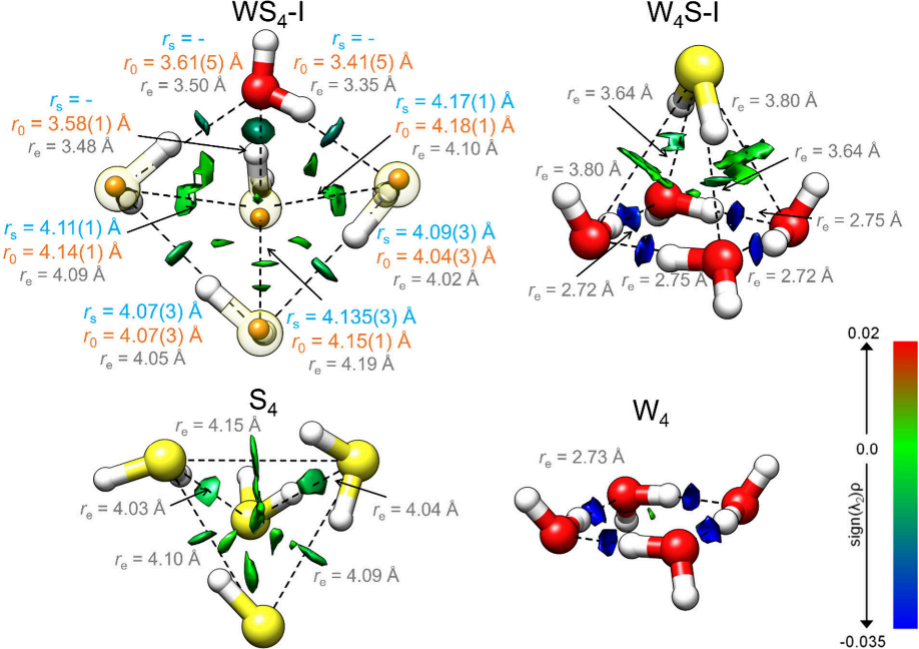
NCI plots^67,68^ (isosurface 0.5) for WS_4_, W_4_S, the hydrogen sulfide (S_4_) and the water tetramers (W_4_), respectively. The larger spheres are the predicted structures at the ωB97X-V/def2-TZVP level of theory (*r*_e_) along with some intermolecular distances. The smaller, solid spheres overlaid with the structure of WS_4_ represent the experimentally determined (*r*_s_) sulfur atom positions obtained from the monoisotopic substitutions in natural abundance. The S···S, S···O, O···S, and O···O distances are given in Å. The strength of the interactions is indicated by the value and sign of the second eigenvalue of the Hessian, λ_2_. Negative values indicate a strong attraction and are depicted in blue, while values close to 0 indicate the presence of weak forces and are illustrated in green.

The WS_4_ pentamer features a distorted square pyramid, with the only water molecule positioned in one of the vertices of the base. The four molecules in the base simultaneously act as hydrogen bond donor and acceptor, closing an 8-membered ring. In addition, the three hydrogen sulfide molecules in the base act as proton donors to the molecule in the apex, which is involved in four simultaneous hydrogen-bonding interactions (three as an acceptor and one as donor). In total, one O–H···S, two S–H···O, and five S–H···S individual interactions can be identified. Notably, the S···S interaction previously described as a plausible attractive force^53–56^ was not identified in the cluster.

In Figure 3, we compare WS_4_ with the homomeric S_4_ tetramer obtained from quantum chemistry. It is worth noting that the hydrogen-bonded network present in the pure tetramer closely resembles that identified in WS_4_. As depicted, the structure of the pure tetramer is slightly distorted to accommodate the water monomer favorably through the formation of the above-mentioned hydrogen bonding topologies. This does not imply that the formation of this cluster occurs sequentially through the addition of water monomers to the structure of the pure tetramer. Instead, it helps to identify the presence and pervasiveness of specific hydrogen-bonded networks as the cluster complexity increases.

The second pentamer, W_4_S, presents a pyramidal shape with a regular square base made of four water units resembling the pure water tetramer (Figure 3). In W_4_S, the hydrogen sulfide unit is located in the apex, binding simultaneously to the four water molecules. The inclusion of hydrogen sulfide does not significantly alter the dominant water tetramer but generates a nonplanar structure that maximizes its interactions with water through two O–H···S and two S–H···O hydrogen bonds. The final polyhedric structure reflects the well-known self-aggregation preference of water, whose hydrogen bond is only slightly perturbed to locate the hydrogen sulfide monomer. Overall, both WS_4_ and W_4_S share the total number of network interactions (eight in total), but of different nature.

A Non-Covalent Interaction (NCI) analysis was performed to visualize the multiple interactions stabilizing the clusters. The plotted surfaces in Figure 3 are based on the reduced density gradient as suggested by Johnson et al.,^67,68^ mapping the spatial distribution, attractive character, and strength of the intermolecular forces in the pentamers. This information, together with structural data, confirms the S–H····S hydrogen bond as a weak interaction.

### Many-Body Decomposition Energy Analysis

To facilitate the interpretation of our findings and provide a rationale for the structures observed in the clusters, we conducted a comprehensive many-body decomposition (MBD) analysis of the interaction energy for each cluster.^69,70^ This analysis yields valuable insights into the strength of specific interactions between molecule pairs, triples, and higher-order groups, as well as their individual contributions to the overall interaction energy. We computed the individual many-body interaction energy components for all monomer sets to isolate each interaction of interest. Additionally, we performed an MBD analysis on the two extreme cases with the same number of monomers, i.e., the pure hydrogen sulfide pentamer (S_5_) and the pure water pentamer (W_5_), respectively. This allowed us to observe the trend for each *n*-body interaction as the relative number of each type of monomers is reversed. The results of this analysis are illustrated in Figure 4, and the complete set of results is reported in the SI (Table S9 and Figure S5). This analysis was performed at the ωB97X/def2-TZVP level of theory for all 31 possible *n*-body (*n* = 1–5) contributions. Subsequently, we will highlight the most relevant findings.

**Figure 4:**
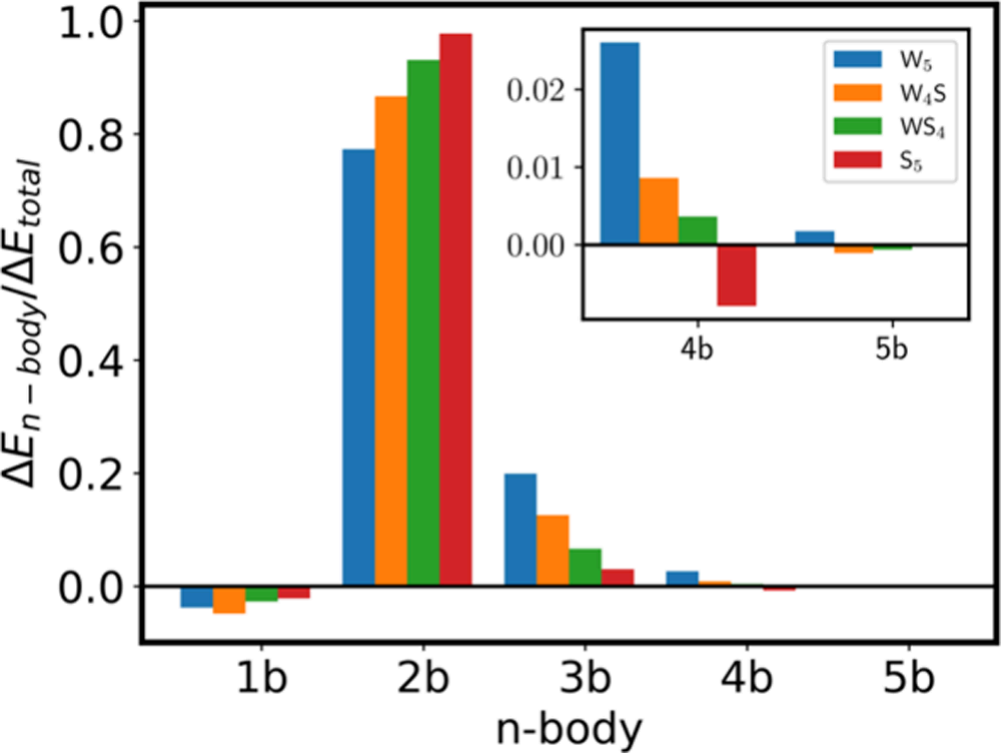
*n*-Body normalized contributions to the total interaction energy for the observed W_4_S (orange) and WS_4_ (green) clusters compared to the pure water pentamer W_5_ (blue) and the pure hydrogen sulfide pentamer S_5_ (red). The two- and three-body contributions are the dominant ones and give the clusters their specific structural features. The pairwise contributions increase significantly as the number of H_2_S moieties increases. The opposite trend is observed for the three-body interactions. The total interaction/four-body interactions ratio shown in the inset becomes negative for the pure S_5_ cluster.

First, we observe that the one-body or deformation energy is in the range of 2–5% of the total energy. This small contribution is expected because the monomers in the cluster are only slightly distorted from their isolated, most stable angular form. Second, the two-body interactions are the main contributors to the global interaction energy, ranging from a 77% for W_5_ to a remarkably higher 98% for the pure S_5_ cluster. Third, the three-body contributions decrease notably as the amount of H_2_S monomers increases in the clusters. In this case, we go from a 20% for W_5_ to an almost negligible 3% for S_5_.

These results have important implications in the structural features of the observed clusters. In pure water clusters, it is accepted that the three-body interactions are one of the main factors contributing to both the preferred three-dimensional geometries and cooperativity, which remarkably increases the strength of a hydrogen bond as the number of water monomers in the network increases.^71^ However, the dramatic decrease in their contribution to the total interaction energy for S_5_ clearly indicates that cooperative effects in the aggregation of H_2_S are considerably less relevant compared to those in water. What is more, the four-body contributions become anticooperative for S_5_ as shown in the Figure 4 inset. All these findings indicate that the three-dimensional structures are predominantly governed by pairwise interactions, optimizing the geometries of individual dimers, which strongly contributes to the shallow PES observed for clusters containing H_2_S and the difficulty encountered for quantum-chemical calculations to explore the structure and energetics of sulfur-centered bonded systems.

## Conclusions

In summary, we used state-of-the-art broadband rotational spectroscopy combined with quantum-chemical calculations to fully characterize the elusive, largely unexplored sulfur-centered hydrogen bonds through the study of two extreme cases of mixed pentamers, i.e., W_4_S and WS_4._ The high sensitivity and resolution of rotational spectroscopy, together with the exquisite sensitivity to the three-dimensional structure and mass distribution of the clusters under study, enabled us to obtain experimental structural parameters with unprecedented accuracy. In addition, we performed a comprehensive MBD energy analysis of the observed clusters together with pure, homodromic aggregates to gain insight into the individual contributions of particular monomer groups to the total interaction energy. Remarkably, we found that the multibody contributions to the total interaction energy are significantly different as the relative proportions of H_2_O–H_2_S vary.

These results help to rationalize the observed structures and contribute to a better understanding of the cooperative forces that, while crucial in water clusters, are much less relevant to the global geometry and energetics in H_2_S-containing clusters. The current results represent a significant advance in elucidating this underexplored area of sulfur-centered hydrogen bonding. They will undoubtedly contribute to better modeling of these important interactions for a variety of research areas. Future investigations of other hydrogen sulfide adducts may contribute to learn about the internal dynamics of the clusters and to understand how the molecular characteristics contribute to their different macroscopic properties compared to water.

## Experimental Methods

The experimental investigation used broadband microwave spectroscopy in the 2–12 GHz region using the chirped-pulse Fourier transform microwave (CP-FTMW) spectrometer COMPACT.^72^ The sample, containing *ca*. 1% of H_2_S in neon, was directed to a gas mixing line permitting the addition of water vapor. The complexes were generated by supersonic expansion of the gas mixture (2.5 bar) through a pulsed injection valve (nozzle diameter 1 mm). A short microwave chirped pulse (4 μs) perpendicular to the gas jet produced a transient broadband electric dipole molecular excitation. The resulting free induction decay of the induced macroscopic dipole moment was recorded in a digital oscilloscope (40 μs) and Fourier-transformed to give the frequency spectrum. Typical transitions have full widths at half-maximum of ca. 50 kHz. A complete description of the theoretical and experimental details is given in the Supporting Information. The observed frequencies of the rotational transitions are given in the SI.

## Supplementary Material



## Notes

The Supporting Information provides a description of the theoretical and experimental methods, theoretical parameters for the considered structures, experimental data for ^34^S isotopologues, many-body decomposition analysis energies; and line lists of observed transitions (PDF)
