# Supplementary material for: Hydrogen Bond Interaction Networks in the Mixed Pentamers of Hydrogen Sulfide and Water
Source: J Am Chem Soc. 2025 May 26;147(22):18576–82. doi: 10.1021/jacs.4c18276 (PMC12147112; doi:10.1021/jacs.4c18276)
Supplement: Supplementary file 1 [file ja4c18276_si_001.pdf]

Supporting Information for:

Hydrogen Bond Interaction Networks in the Mixed Pentamers of Hydrogen Sulfide and Water

Pablo Pinacho,[a],[b] Cristóbal Pérez,[a],[b],* Marcel Stahn,[c] Rizalina T. Saragi,[b],[d] Andreas Hansen,[c] Stefan Grimme,[c] Alberto Lesarri,[b],* and Melanie Schnell.[a],[e],*

[a] Deutsches Elektronen-Synchrotron DESY, Notkestr. 85, 22607 Hamburg (Germany)

[b] Departamento de Química Física y Química Inorgánica, Facultad de Ciencias – I.U. CINQUIMA, Universidad de Valladolid, Paso Belén 7, 47011 Valladolid (Spain)

[c] Mulliken Center for Theoretical Chemistry, Institut für Physikalische und Theoretische Chemie, Rheinische Friedrich-Wilhelms-Universität Bonn, Beringstraße 4, 53115 Bonn (Germany)

[d] Chemical Sciences and Engineering Division, Argonne National Laboratory, Lemont, Illinois 60439, United States

[e] Institut für Physikalische Chemie, Christian-Albrechts-Universität zu Kiel, Max-Eyth-Str. 1, 24118 Kiel (Germany)

KEYWORDS. Hydrogen sulfide - Molecular clusters - Hydrogen bonding - Molecular Structure - Rotational spectroscopy.

Table of Contents:

**Theoretical and Experimental Details**	S2-S3
**Experimental Structure**	S3
**Table S1**. Theoretical parameters for the lowest-energy $(H_2O)\cdots(H_2S)_4$, (WS_4) conformers	S4
**Table S2**. Theoretical parameters for the lowest-energy $(H_2O)_4\cdots(H_2S)$, (W_4S) conformers	S4
**Figure S1.** Experimental relative intensities for W_4S and WS_4.	S5
**Table S3.** Theoretical parameters for the family of conformers WS_4-I	S6
**Figure S2.** Theoretical structures for the family of conformers WS_4-I	S6
**Table S4.** Rotational constants for the parent and the observed isotopologues for WS_4-I	S7
**Figure S3.** Experimental spectrum highlighting transitions for ^{34}S isotopologues for WS_4-I	S7
**Table S5.** Experimental (r_s, r_0) and theoretical r_e inertial axis coordinates for WS_4-I	S8
**Table S6.** Results from the r_0 fit for WS_4-I.	S8
**Table S7.** Experimental (r_s, r_0) and theoretical r_e distances for WS_4-I	S9
**Table S8.** Theoretical (r_e) hydrogen bond distances for WS_4-I and W_4S-I	S9
**Figure S4.** Molecular structures for WS_4-I and W_4S-I with complete atom labelling	S9
**Table S9.** Many-body decomposition analysis in W_5, S_5, W_4S, and WS_4	S10
**Figure S5.** Fragment definition for many-body analysis for W_5, S_5, W_4S, and WS_4	S11
**Table S10**. Observed frequencies and residuals for the parent species of WS_4-I	S12-S14
**Tables S11-S14**. Observed frequencies and residuals for the ^{34}S isotopologues of WS_4-I	S15-S18
**Table S15**. Observed frequencies and residuals for the parent species of W_4S-I	S19
**Tables S16-S18.** Theoretical coordinates for WS_4 conformers	S20
**Tables S19-S21.** Theoretical coordinates for W_4S conformers	S21

Theoretical and Experimental Details

The analysis of the spectrum was guided by theoretical computations. The potential conformations for each ensemble were investigated independently within an energy window of 10 kJ·mol^{-1} using the CREST and ENSO tools[1,2] and the xTB program.[3,4] The resulting structures were checked for duplicates, however, this step proved to be challenging due to the high degrees of freedom for those weakly-bounded complexes. All the conformers with a 1% similarity in the rotational constants to a previous structure were removed while keeping the rest. The remaining conformers were optimized at the PBE0-D4/def2-QZVPP level of theory. Accurate relative energies were computed for that ensemble of conformers using an explicitly correlated local coupled cluster method with single, double, and triple perturbative excitations and an extended basis set with diffuse functions (PNO-LCCSD(T)-F12b/aug-cc-pVTZ+D (H=cc-pVTZ; denoted AVTZ' in the following)[5-7] with tight domain settings as implemented in Molpro V. 2022.3).[8,9] In a subsequent stage, all the conformers below 2 kJ·mol^{-1} were re-optimized using ORCA, version 5.0[10,11] including zero-point energies from harmonic frequencies at the B3LYP-D3(BJ)/def2-TZVP, ωB97X-V/def2-TZVP, and MP2/aug-cc-pVTZ levels of theory. Additional harmonic frequency calculations were performed using Gaussian 16 A.03[12] at ωB97X-D/def2-TZVP. The predictions at ωB97X-V/def2-TZVP seem to be in better agreement with the experimental values than the other two levels of theory. This fact could be due to beneficial error cancelation.

The Non-Covalent Interactions (NCIs) were identified and visualized by performing a new set of calculations to obtain the value and sign of the second eigenvalue of the Hessian, λ_2. In the NCI plots, the interactions are illustrated as colored surfaces, in which blue (negative values of λ_2) is associated with attractive interactions, green (values of λ_2 close to 0) with weak interactions, and red (positive values of λ_2) with repulsions. The data for the NCI plots were generated with the Multiwfn[13] software and visualized with Chimera.[14]

[1] Pracht, P.; Bohle, F.; Grimme, S. Automated exploration of the low-energy chemical space with fast quantum chemical methods. *Phys. Chem. Chem. Phys.* **2020**, *22*, 7169-7192.
[2] Salthammer, T.; Grimme, S.; Stahn, M.; Hohm, U.; Palm, W.-U. Quantum Chemical Calculation and Evaluation of Partition Coefficients for Classical and Emerging Environmentally Relevant Organic Compounds. *Environ. Sci. Tecnol.* **2022**, *56*, 379-391.
[3] Grimme, S.; Bannwarth, C.; Shushkov, P. A Robust and Accurate Tight-Binding Quantum Chemical Method for Structures, Vibrational Frequencies, and Noncovalent Interactions of Large Molecular Systems Parametrized for All spd-Block Elements (Z = 1-86). *J. Comput. Theory Chem.* **2017**, *13*, 1989-2009.
[4] Bannwarth, C.; Ehlert, S.; Grimme, S. GFN2-xTB—An Accurate and Broadly Parametrized Self-Consistent Tight-Binding Quantum Chemical Method with Multipole Electrostatics and Density-Dependent Dispersion Contributions. *J. Chem. Theory Comput.* **2019**, *15*, 3, 1652-1671.
[5] Ma, Q.; Schwilk, M.; Köppl, C.; Werner, H. J. Scalable Electron Correlation Methods. 4. Parallel Explicitly Correlated Local Coupled Cluster with Pair Natural Orbitals (PNO-LCCSD-F12). *J. Chem. Theor. Comput.* **2017**, *13*, 4871–4896.
[6] Ma, Q.; Werner, H. J. Explicitly correlated local coupled-cluster methods using pair natural orbitals. *Comput. Mol. Sci.* **2018**, *8*, e1371.
[7] Dunning, T. H. Gaussian basis sets for use in correlated molecular calculations. I. The atoms boron through neon and hydrogen. *J. Chem. Phys.* **1989**, *90*, 1007–1023.
[8] Werner, H. J.; *et al.* The Molpro quantum chemistry package. *J. Chem. Phys.* **2020**, *152*, 144107.
[9] Werner, H. J.; *et al.* "Molpro, 2022.3 , a package of ab initio programs".
[10] Neese, F. The ORCA program system. *Wiley Interdiscip. Rev. Comput. Mol. Sci.* **2012**, *2*, 73–78.
[11] Neese, F. Software update: The ORCA program system—Version 5.0. *Wiley Interdiscip. Rev. Comput. Mol. Sci.* **2022**, 12, e1606.
[12] Frisch, M. J.; *et al.*, Gaussian Inc., Wallingford CT, **2016**.
[13] Lu, T.; Chen, F. Multiwfn: A multifunctional wavefunction analyzer. *J. Comput. Chem.* **2012**, *33*, 580-592.
[14] Pettersen, E. F.; Goddard, T. D.; Huang, C. C.; Couch, G. S.; Greenblatt, D. M.; Meng, E. C.; Ferrin, T. E. UCSF Chimera--a visualization system for exploratory research and analysis. *J. Comput. Chem.* **2004**, *25*, 1605-1612.

The experimental spectra were recorded using the chirped-pulse Fourier transform microwave (CP-FTMW)[15] spectrometer COMPACT in Hamburg.[16] The spectrometer can cover the 2-18 GHz frequency region in three ranges; 2-8, 8-12, and 12-18 GHz. The sample of H_2S (1% in neon) was directed to the pulsed nozzle passing by a homemade gas set-up which allowed to open or close the gas line containing water vapor. The amount of water in the gas was controlled by the opening of its valve, keeping the total pressure of the gas mixture around 2.5 bar. A supersonic expansion through the small diameter nozzle (1 mm) into the vacuum chamber (10^{-7} mbar) and with a duration of 1.0 *ms* generated the complexes in isolated conditions. After a small delay, a short microwave pulse of 4 *μs* was generated, amplified, and broadcasted into the vacuum chamber by a horn antenna.
The microwave pulse consists of a linear sweep covering the respective frequency range and inducing a macroscopic polarization of the ensemble. Two different electronic arrangements were employed for recording the spectra in the 2-8 and 8-12 frequency regions, using different amplifiers for the excitation part, keeping the rest of the electronic devices. Once the microwave excitation pulse ceases, and after a small delay to protect the detection devices, the molecular Free Induction Decay (FID) signal is registered, amplified, and recorded in a digital oscilloscope. The FID was registered in the time domain, and the application of the Fourier transform gave the spectra in the frequency domain. Additionally, a fast frame set-up[17] was employed allowing for the repetition of eight emission-detection cycles per supersonic gas pulse. The pulsed valve worked at 8 Hz, thus resulting in a total repetition rate of 64 Hz. For the spectrum between 2 and 8 GHz, a total of 4.4 million FIDs were averaged, while between 8-12 GHz, 5.6 million FIDs were co-added. Many of the experimental lines in the spectrum showed a fine structure probably arising from large amplitude motions, as observed in previous complexes involving H_2S. Surprisingly, the transitions of the two species reported here did not present a splitting.

Experimental Structure

Two approaches have been employed to exploit the information of the observed mono-substituted isotopologues (^{34}S, natural abundance ≈ 4%) and to derive experimental structures. In the first one, which gives the substitution structure (r_S), the Kraitchman equations are solved.[18] This method assumes that there is no change in the interatomic distances with the isotopic substitution and allows to build the experimental structure atom by atom from their coordinates in the principal inertial axis system. The r_S structure gives an accurate description of the molecule, but it has several drawbacks. For atoms near any of the principal axes or planes, the solution of the Kraitchman equations may result in imaginary values for the coordinate. In addition, the Kraitchman equations only give the value of the coordinate, but not its sign, which has to be assigned from comparison with other methods or theoretical predictions.

The second method, that gives the so-called effective structure (r_0), is based on a least-squares fit of selected structural parameters (bond distances, bond angles, or dihedral angles) to reproduce the experimental rotational constants in the ground vibrational state (v_0).[19,20] This method can be applied even without the observation of isotopologues, however the more experimental information, the more parameters can be fit. Normally, not all single-substituted isotopologues are observed; for most of the molecules only the heavy atoms are observed, while the distances of the hydrogen atoms are fixed to the theoretical values. Thus, it is not possible to obtain a complete r_0 structure determination, but rather a partial effective structure fitting the distances and angles of the heavy atom skeleton.

---

[15] Brown, G. G.; Dian, B. C.; Douglass, K. O.; Geyer, S. M.; Shipman, S. T.; Pate, B. H. A broadband Fourier transform microwave spectrometer based on chirped pulse excitation. *Rev. Sci. Instrum.* **2008**, *79*, 053103.
[16] Schmitz, D.; Alvin Shubert, V.; Betz, T.; Schnell, M. Multi-resonance effects within a single chirp in broadband rotational spectroscopy: The rapid adiabatic passage regime for benzonitrile. *J. Mol. Spectrosc.* **2012**, *280*, 77–84.
[17] Pérez, C.; Lobsiger, S.; Seifert, N. A.; Zaleski, D. P.; Temelso, B.; Shields, G. C.; Kisiel, Z.; Pate, B. H. Broadband Fourier Transform Rotational Spectroscopy for Structure Determination: The Water Heptamer. *Chem. Phys. Lett.* **2013**, *571*, 1–15.
[18] Kraitchman, J. Determination of Molecular Structure from Microwave Spectroscopic Data. *Am. J. Phys.*, **1953**, *21*, 17–24.
[19] Rudolph, H. D.; Demaison, J. Determination of the Structural Parameters from the Inertial Moments, in *Equilibrium Molecular Structures* (Ed.: Demaison, J; Boggs, J. E.; Csaszar, A. G.), CRC Press, Boca Raton, Fl, **2011**, pp. 125–158.
[20] Kisiel, Z. Least-squares mass-dependence molecular structures for selected weakly bound intermolecular clusters. *J. Mol. Spectrosc.*, **2003**, *218*, 58–67.

Table S1. Theoretical parameters for the lowest-energy $(H_2O)\cdots(H_2S)_4$, (WS_4) conformations.

	B3LYP-D3(BJ)/def2-TZVP			ωB97X-V/def2-TZVP			MP2/aug-cc-pVTZ		
	WS_4-I	WS_4-II	WS_4-III	WS_4-I	WS_4-II	WS_4-III	WS_4-I	WS_4-II	WS_4-III
A /MHz[a]	916	862	1143	875	824	1077	952	923	1183
B /MHz	714	630	531	684	596	516	730	659	547
C /MHz	606	607	477	576	585	460	623	625	496
$\lvert\mu_a\rvert$ /D	1.1	1.3	0.9	1.2	1.5	1.0	1.1	1.0	0.9
$\lvert\mu_b\rvert$ /D	2.9	0.6	1.2	3.0	0.8	1.4	2.6	2.4	1.2
$\lvert\mu_c\rvert$ /D	0.2	2.4	0.1	0.3	2.4	0.1	0.3	0.9	0.1
ΔE /cm^{-1}	0.0	69.5	168.1	0.0	98.8	117.0	0.0	46	415.7
ΔE /kJ·mol^{-1}	0.0	0.8	2.0	0.0	1.2	1.4	0.0	0.6	5.0
ΔE_{ZPE} /cm^{-1}	0.0	52.0	229.2	113.4	0.0	270.9	0.0	74	368.7
ΔE_{ZPE} /kJ·mol^{-1}	0.0	0.6	2.7	1.4	0.0	3.2	0.0	0.9	4.4

	PNO-LCCSD(T)-F12b/TIGHT/AVTZ'		
	WS_4-I	WS_4-II	WS_4-III
ΔE_{LCC} /cm^{-1}	0.0	111.9	97.9
ΔE_{LCC} /kJ·mol^{-1}	0.0	1.3	1.2

[a] Rotational constants (A, B, C), electric dipole-moment components (μ_α, α = a, b, or c), relative energies (ΔE), relative energies including the zero-point energy correction (ΔE_{ZPE}), and relative energies obtained with PNO-LCCSD(T)-F12 (ΔE_{LCC}).

Table S2. Theoretical parameters for the lowest-energy $(H_2O)_4\cdots(H_2S)$, (W_4S) conformations.

	B3LYP-D3(BJ)/def2-TZVP			ωB97X-V/def2-TZVP			MP2/aug-cc-pVTZ		
	W_4S-I	W_4S-II	W_4S-III	W_4S-I	W_4S-II	W_4S-III	W_4S-I	W_4S-II	W_4S-III
A /MHz[a]	1845	2437	2054	1823	2571	2042	1847	2584	2078
B /MHz	1432	1055	1321	1410	975	1292	1460	1022	1333
C /MHz	1377	916	1291	1352	835	1259	1413	877	1297
$\lvert\mu_a\rvert$ /D	1.0	1.2	2.7	1.1	1.2	2.7	1.0	1.1	2.4
$\lvert\mu_b\rvert$ /D	0.0	1.0	2.5	0.0	1.0	2.6	0.0	1.0	2.4
$\lvert\mu_c\rvert$ /D	0.0	0.6	0.9	0.0	0.6	0.3	0.0	0.6	0.4
ΔE /cm^{-1}	0.0	98.2	143.3	0.0	168.6	152.8	0.0	176.3	115.6
ΔE /kJ·mol^{-1}	0.0	1.2	1.7	0.0	2.0	1.8	0.0	2.1	1.4
ΔE_{ZPE} /cm^{-1}	0.0	302.7	318.3	0.0	164.2	116.8	0.0	393.1	299.5
ΔE_{ZPE} /kJ·mol^{-1}	0.0	3.6	3.8	0.0	2.0	1.4	0.0	4.7	3.6

	PNO-LCCSD(T)-F12b/TIGHT/AVTZ'		
	W_4S-I	W_4S-II	W_4S-III
ΔE_{LCC} /cm^{-1}	0.0	83.9	118.9
ΔE_{LCC} /kJ·mol^{-1}	0.0	1.0	1.4

[a] Rotational constants (A, B, C), electric dipole-moment components (μ_α, α = a, b, or c), relative energies (ΔE), relative energies including the zero-point energy correction (ΔE_{ZPE}), and relative energies obtained with PNO-LCCSD(T)-F12 (ΔE_{LCC}).

Figure S1. The upper panel presents selected lines for WS_4 showing the relative intensity ratio for *a*-, *b*-, and *c*-type transitions. The lower panel presents selected lines for W_4S showing the intensity of the observed a-type transitions.

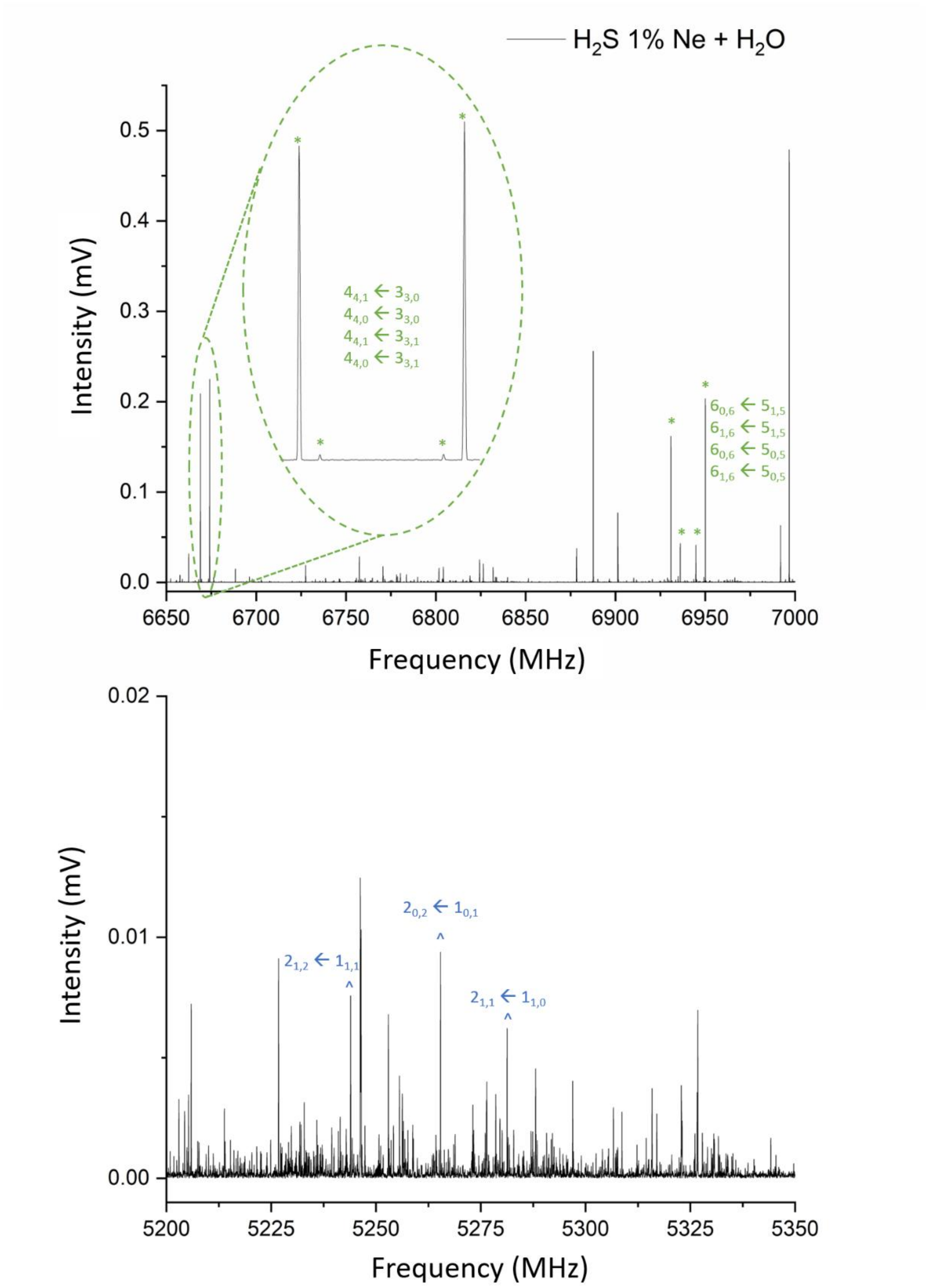

Table S3. Theoretical parameters for different orientations of the hydrogen atoms for the family of conformers WS_4-I at the ωB97X-V/def2-TZVP level of theory.

	WS_4-I a	WS_4-I b	WS_4-I c	WS_4-I d	WS_4-I e	WS_4-I f	WS_4-I g	WS_4-I h*
A /MHz[a]	875	858	878	883	899	901	856	880
B /MHz	684	695	674	696	716	693	695	700
C /MHz	576	567	579	577	587	589	586	591
$\|\mu_a\|$ /D	1.2	1.2	0.9	2.1	2.0	0.5	2.1	1.9
$\|\mu_b\|$ /D	3.0	2.0	2.5	2.3	1.6	2.0	1.8	1.6
$\|\mu_c\|$ /D	0.3	1.1	1.0	0.8	1.8	1.6	2.4	3.2
ΔE /cm^{-1}	0.0	129.4	198.9	212.9	343.6	350.0	372.2	557.4
ΔE /kJ·mol^{-1}	0.0	1.5	2.4	2.5	4.1	4.2	4.5	6.7
ΔE_{ZPE} /cm^{-1}	0.0	123.6	183.9	202.4	336.7	323.6	339.4	316.8
ΔE_{ZPE} /kJ·mol^{-1}	0.0	1.5	2.2	2.4	4.0	3.9	4.1	3.8

[a] Rotational constants (A, B, C), electric dipole-moment components (μ_α, α = a, b or c), relative energy (ΔE), and relative energy including the zero-point energy correction (ΔE_{ZPE}). * Contains an imaginary frequency.

Figure S2. Theoretical structures for the different orientations of the hydrogen atoms for the family of conformers WS_4-I at the ωB97X-V/def2-TZVP level of theory. Conformers are sorted based on their relative stabilization energies. The zero-point corrected energies are given relative to the most stable conformation.

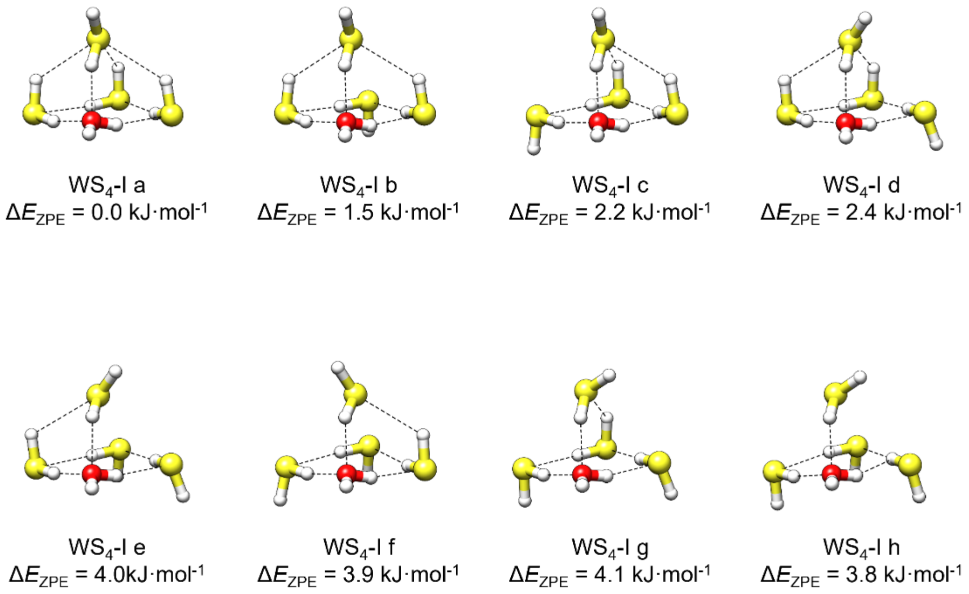

Table S4. Rotational constants for the main isotopologue (parent) and the observed ^{34}S monosubstituted isotopologues of WS_4-I. Atom labeling is provided in **Figure 2** and **Figure S4**.

	Parent	$^{34}S_1$	$^{34}S_2$	$^{34}S_3$	$^{34}S_4$
A /MHz[a]	865.25110(11)[b]	849.56224(47)	863.53716(30)	845.96025(36)	863.17555(40)
B /MHz	657.994990(95)	651.17537(64)	643.79366(27)	657.88686(48)	643.57771(49)
C /MHz	564.323353(88)	562.58394(29)	554.51157(19)	556.12639(26)	554.48239(30)
Δ_J /kHz	0.53003(57)	0.5066(78)	0.5180(27)	0.5311(52)	0.5154(48)
Δ_{JK} /kHz	-1.3627(11)	-1.234(33)	-1.374(14)	-1.377(20)	-1.362(22)
Δ_K /kHz	2.9068(12)	2.704(26)	2.925(12)	2.824(15)	2.927(19)
δ_J /kHz	0.14284(18)	0.1223(39)	0.1355(13)	0.1484(25)	0.1372(25)
δ_K /kHz	-0.2598(20)	-0.336(34)	-0.236(22)	-0.280(25)	-0.271(38)
N	260	58	67	63	62
σ /kHz	5.4	4.9	3.6	4.1	4.5

[a] Rotational constants (A, B, C), quartic centrifugal distortion constants (Δ_J, Δ_{JK}, Δ_K, δ_J, and δ_K), number of fitted transitions (N), root-mean square deviation of the fit (σ). [b] Standard error in parentheses in units of the last digit.

Figure S3. Zoom-in of the experimental spectrum highlighting selected transitions for the ^{34}S isotopologues of WS_4-I. Atom labeling is provided in **Figure 2** and **Figure S4**.

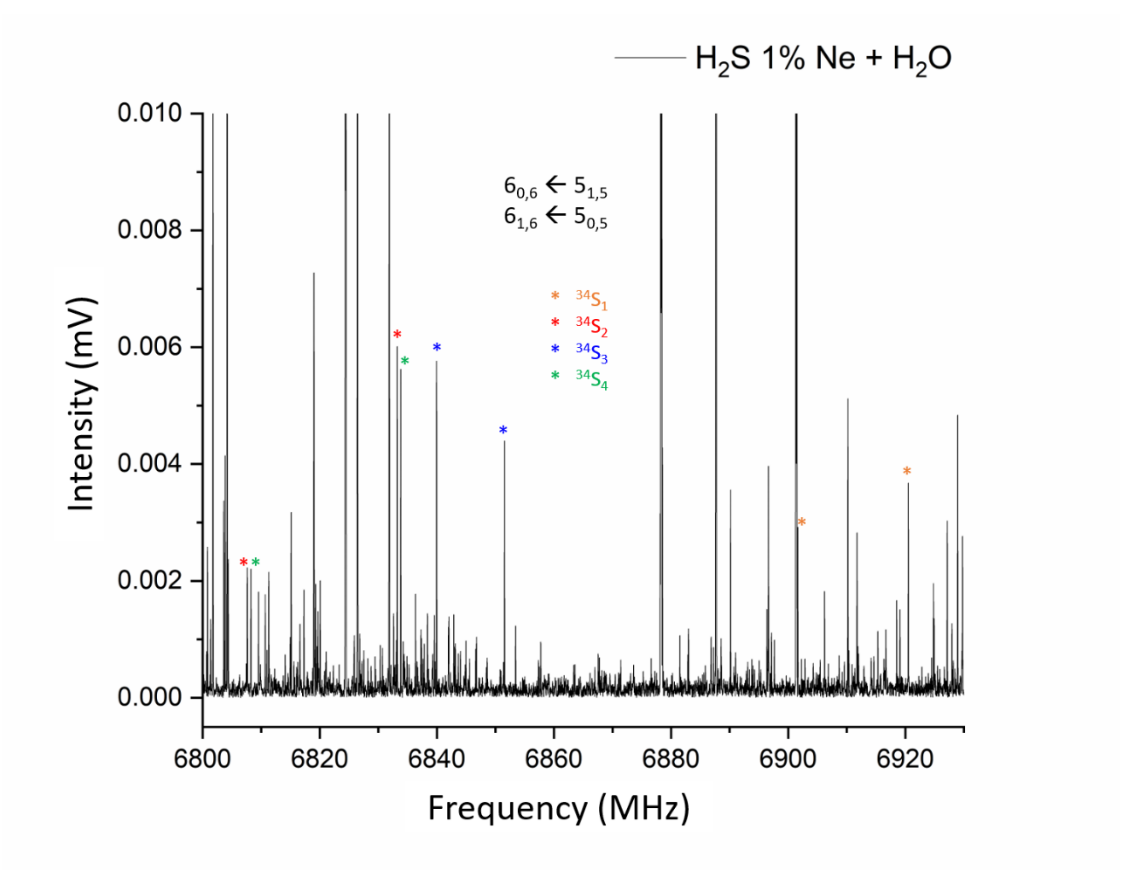

Table S5. Experimental r_s, r_0, and theoretical r_e (B3LYP-D3(BJ)/def2-TZVP, ωB97X-V/def2-TZVP, and MP2/aug-cc-pVTZ) inertial axis coordinates (values in Å) for the S_1, S_2, S_3, S_4, and O_5 atoms for WS_4-I. Atom labeling is provided in **Figure 2** and **Figure S4**.

	S_1		
	a	b	c
$r_{e\text{-B3LYP}}$	-0.05	-1.28	1.91
$r_{e\text{-ωB97X-V}}$	-0.06	-1.32	1.93
$r_{e\text{-MP2}}$	-0.04	-1.22	1.86
r_s	-0.08(2)	-1.145(1)	2.0411(7)
r_0	-0.061(3)	-1.15(1)	2.047(4)
	S_2		
	a	b	c
$r_{e\text{-B3LYP}}$	-2.71	-0.05	-0.85
$r_{e\text{-ωB97X-V}}$	-2.78	-0.09	-0.86
$r_{e\text{-MP2}}$	-2.70	-0.05	-0.84
r_s	-2.8302(5)	-0.159(9)	-0.851(2)
r_0	-2.840(4)	-0.14(3)	-0.85(1)
	S_3		
	a	b	c
$r_{e\text{-B3LYP}}$	-0.02	2.50	0.40
$r_{e\text{-ωB97X-V}}$	-0.02	2.59	0.43
$r_{e\text{-MP2}}$	-0.02	2.45	0.42
r_s	-0.04(4)	2.5874(6)	0.262(6)
r_0	-0.02(3)	2.591(5)	0.25(1)
	S_4		
	a	b	c
$r_{e\text{-B3LYP}}$	2.72	-0.03	-0.71
$r_{e\text{-ωB97X-V}}$	2.79	-0.04	-0.73
$r_{e\text{-MP2}}$	2.70	-0.04	-0.71
r_s	2.8284(5)	-0.13(1)	-0.776(2)
r_0	2.840(3)	-0.08(3)	-0.76(1)
	O_5		
	a	b	c
$r_{e\text{-B3LYP}}$	0.12	-1.91	-1.47
$r_{e\text{-ωB97X-V}}$	0.13	-1.93	-1.48
$r_{e\text{-MP2}}$	0.11	-1.91	-1.45
r_s	-	-	-
r_0	0.16(5)	-2.08(2)	-1.402(8)

Table S6. Results from the r_0 fit for WS_4-I showing the reproduced rotational constants for the ground state and the statistical parameters of the fit.

	Experimental	r_0
A /MHz[a]	865.25110(11)	865.26540
B /MHz	657.994990(95)	658.03615
C /MHz	564.323353(88)	564.35903
χ^2	0.0347	
Fit deviation	0.0658	

Table S7. Experimental (r_s and r_0) and theoretical (r_e, B3LYP-D3(BJ)/def2-TZVP, ωB97X-V/def2-TZVP, and MP2/aug-cc-pVTZ) distances between the heavy atoms for WS_4-I. Atom labeling is provided in **Figure 2** and **Figure S4**.

Distances (Å)	$r_{e\text{-B3LYP}}$	$r_{e\text{-}\omega\text{B97X-V}}$	$r_{e\text{-MP2}}$	r_s	r_0
$S_1 \cdots S_2$	4.02	4.09	3.97	4.11(1)	4.14(1)[a]
$S_1 \cdots S_3$	4.08	4.19	3.95	4.135(3)	4.15(1)[a]
$S_1 \cdots S_4$	4.02	4.10	3.94	4.17(1)	4.18(1)
$S_1 \cdots O_5$	3.43	3.48	3.39	-	3.58(1)[a]
$S_2 \cdots S_3$	3.92	4.05	3.88	4.07(3)	4.07(3)
$S_2 \cdots S_4$	5.44	5.57	5.40	5.6592(7)	5.68(5)[a]
$S_2 \cdots O_5$	3.44	3.50	3.42	-	3.61(5)[a]
$S_3 \cdots S_4$	3.89	4.02	3.85	4.09(3)	4.04(3)
$S_3 \cdots O_5$	4.79	4.91	4.74	-	4.96(2)[a]
$S_4 \cdots O_5$	3.30	3.35	3.27	-	3.41(5)

[a] Derived parameter obtained from the atomic coordinates resulting from the least-squares fit.

Table S8. Theoretical (r_e, B3LYP-D3(BJ)/def2-TZVP, ωB97X-V/def2-TZVP, and MP2/aug-cc-pVTZ) hydrogen bond distances for WS_4-I and W_4S-I. Atom labeling in **Figure 2** and **Figure S4**.

	WS_4-I		
Distances (Å)	B3LYP	ωB97X-V	MP2
$S_1 \cdots H_9$	3.03	3.13	3.00
$S_1 \cdots H_{11}$	3.14	3.27	3.00
$S_1 \cdots H_{13}$	3.01	3.11	2.93
$S_2 \cdots H_{10}$	2.57	2.72	2.54
$S_3 \cdots H_{12}$	2.56	2.70	2.53
$S_4 \cdots H_{14}$	2.36	2.42	2.33
$O_5 \cdots H_6$	2.16	2.21	2.12
$O_5 \cdots H_8$	2.12	2.18	2.11
	W_4S-I		
Distances (Å)	B3LYP	ωB97X-V	MP2
$S_1 \cdots H_9$	3.40	3.43	3.35
$S_1 \cdots H_{13}$	3.40	3.43	3.35
$O_2 \cdots H_{14}$	1.73	1.75	1.73
$O_3 \cdots H_7$	2.34	2.37	2.29
$O_3 \cdots H_8$	1.78	1.80	1.78
$O_4 \cdots H_{10}$	1.73	1.75	1.73
$O_5 \cdots H_6$	2.34	2.37	2.29
$O_5 \cdots H_{12}$	1.78	1.80	1.78

Figure S4. Molecular structures for WS_4-I and W_4S-I with complete atom labelling.

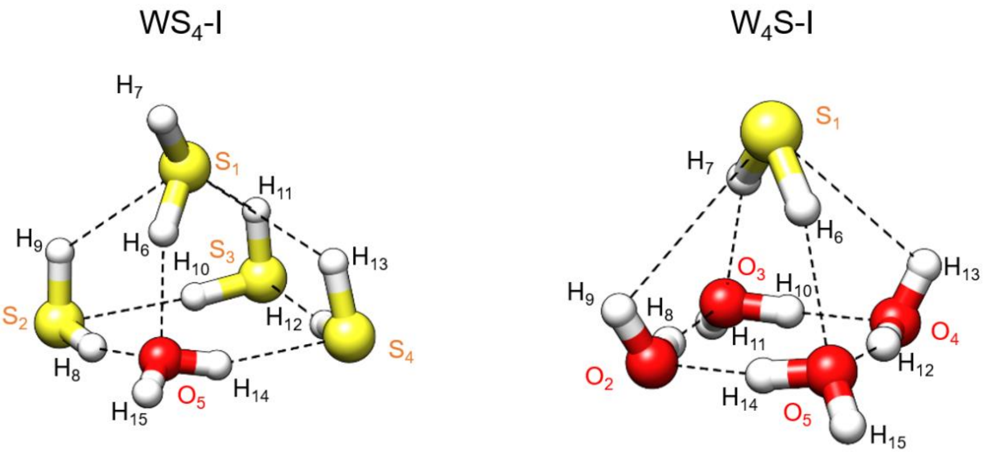

Table S9. Many-body decomposition analysis of the interactions in the water pentamer (W_5), the mixed pentamers W_4S and WS_4, and the hydrogen sulfide pentamer (S_5). All the energies are given in kJ·mol^{-1}. The n-body fragments for the four clusters are defined in **Figure S4**.

	W_5	W_4S	WS_4	S_5
**Individual one body**				
Fragment 1	1.47	1.70	0.92	0.33
Fragment 2	1.46	2.85	0.33	0.39
Fragment 3	1.51	2.44	0.28	0.18
Fragment 4	1.45	1.48	0.51	0.36
Fragment 5	1.51	0.15	0.42	0.22
**Sum 1b (ΔE_{1b})**	**7.39**	**8.62**	**2.46**	**1.47**
**Individual two body**				
Fragment 1-2	-25.42	-26.14	-15.77	-6.70
Fragment 1-3	-5.71	-25.95	-13.71	-8.03
Fragment 1-4	-5.25	-7.30	-15.33	-8.18
Fragment 1-5	-25.31	-4.06	-3.42	-7.44
Fragment 2-3	-22.51	-8.94	-4.91	-4.97
Fragment 2-4	-5.51	-25.58	-1.74	-7.80
Fragment 2-5	-5.23	-12.03	-7.99	-8.92
Fragment 3-4	-25.39	-26.45	-8.41	-8.50
Fragment 3-5	-5.38	-10.00	-4.92	-0.08
Fragment 4-5	-25.30	-8.18	-7.85	-6.27
**Sum 2b (ΔE_{2b})**	**-151.01**	**-154.61**	**-84.06**	**-66.90**
**Individual three body**				
Fragment 1-2-3	-6.02	-6.78	2.85	0.57
Fragment 1-2-4	-1.54	-6.51	-1.90	0.92
Fragment 1-2-5	-6.29	2.32	-1.75	-1.23
Fragment 1-3-4	-1.47	-6.26	-2.93	-1.64
Fragment 1-3-5	-1.59	-1.47	-0.48	0.85
Fragment 1-4-5	-6.33	1.07	-1.77	0.54
Fragment 2-3-4	-6.19	-6.60	0.42	-0.07
Fragment 2-3-5	-1.57	1.10	0.54	-0.67
Fragment 2-4-5	-1.44	-2.56	-1.29	-0.79
Fragment 3-4-5	-6.41	3.31	0.37	-0.54
**Sum 3b (ΔE_{3b})**	**-38.85**	**-22.38**	**-5.94**	**-2.06**
**Individual four body**				
Fragment 1-2-3-4	-1.05	-2.65	0.21	0.20
Fragment 1-2-3-5	-0.99	0.24	0.29	0.11
Fragment 1-2-4-5	-0.99	0.17	-0.85	0.17
Fragment 1-3-4-5	-1.03	0.35	-0.10	0.18
Fragment 2-3-4-5	-1.03	0.36	0.12	-0.12
**Sum 4b (ΔE_{4b})**	**-5.09**	**-1.53**	**-0.33**	**0.54**
**Individual five body**				
**Sum 5b (ΔE_{5b})**	**-0.34**	**0.19**	**0.06**	**0.01**
Interaction energy ($\Delta E_{n\text{-}body}$)	-195.28	-178.34	-90.28	-68.40
Binding energy (BE)	-187.89	-169.72	-87.83	-66.93
$(\Delta E_{1b})/\Delta E$	-0.04	-0.05	-0.03	-0.02
$(\Delta E_{2b})/\Delta E$	0.77	0.87	0.93	0.98
$(\Delta E_{3b})/\Delta E$	0.20	0.13	0.07	0.03
$(\Delta E_{4b})/\Delta E$	0.03	0.01	0.00	-0.01
$(\Delta E_{5b})/\Delta E$	0.00	0.00	0.00	0.00

The binding energy is calculated as the difference between the energy of the cluster and its individual monomers in their most stable isolated conformation. The interaction energy corresponds to the energy of the cluster minus the individual energies of the corresponding monomers in their conformation in the cluster. This allows us to calculate the interaction energy as a summation of deformation energies (one-body) and two-, three-, four-body, etc contributions.

Figure S5. Definition of the n-body fragments for the many-body decomposition analysis in (a) the water pentamer (W_5), (b) the mixed pentamer W_4S, (c) the mixed pentamer WS_4, and (d) the hydrogen sulfide pentamer (S_5).

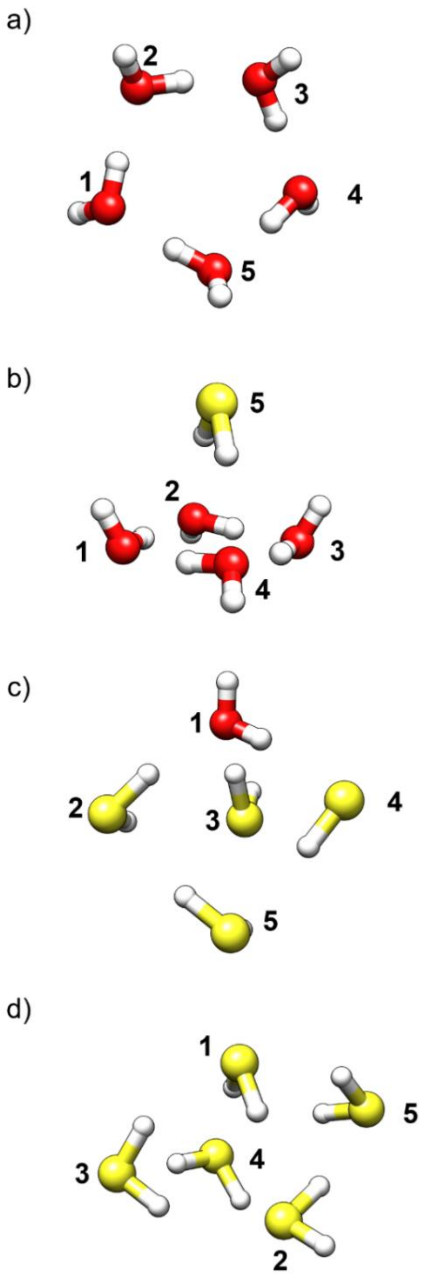

Table S10. Observed frequencies (MHz) and residuals (kHz) for the parent species of WS_4-I for $J'K'_aK'_c \leftarrow J''K''_aK''_c$ transitions.

J'	K'_a	K'_c	J''	K''_a	K''_c	Obs.	Res.
2	0	2	1	1	1	2212.0964	3.1
2	1	2	1	1	1	2350.9564	-0.6
2	0	2	1	0	1	2419.3490	-0.2
2	1	1	1	1	0	2538.2904	-2.8
2	1	2	1	0	1	2558.2119	-1.1
2	1	1	1	0	1	2839.2191	-1.6
2	2	1	1	1	0	3160.0453	-0.8
3	1	2	2	2	1	3167.0588	-1.1
2	2	0	1	1	1	3278.9868	-1.3
3	0	3	2	1	2	3437.1089	0.9
3	1	3	2	1	2	3512.2776	0.0
3	0	3	2	0	2	3575.9727	0.9
4	2	3	3	3	0	3580.2229	-4.7
3	1	3	2	0	2	3651.1409	-0.4
3	2	2	2	2	1	3666.9271	-3.3
3	2	1	2	2	0	3757.8542	-2.3
3	1	2	2	1	1	3788.8118	-0.9
4	2	2	3	3	1	3886.5475	-14.1
3	2	2	2	1	1	4288.6849	1.6
5	3	3	4	4	0	4417.2631	2.2
4	1	3	3	2	2	4511.3369	-3.4
5	3	2	4	4	1	4526.1782	-1.4
4	0	4	3	1	3	4627.6354	-0.9
4	1	4	3	1	3	4661.8964	0.8
5	2	4	4	3	1	4681.3352	1.1
3	2	1	2	1	2	4685.8881	0.4
4	0	4	3	0	3	4702.8062	0.3
4	1	4	3	0	3	4737.0652	0.1
4	2	3	3	2	2	4869.1661	-0.7
3	3	1	2	2	0	4926.1481	0.7
4	3	2	3	3	1	4929.1710	-9.1
3	3	0	2	2	0	4930.6042	4.9
3	3	1	2	2	1	4951.4160	-1.8
4	3	1	3	3	0	4954.4811	-1.1
3	3	0	2	2	1	4955.8669	-2.8
4	1	3	3	1	2	5011.2089	-1.9
4	2	2	3	2	1	5054.8489	-3.6
6	4	3	5	5	0	5178.4104	-4.2
6	4	2	5	5	1	5205.2018	-9.9
5	2	3	4	3	2	5296.9360	0.6
4	2	3	3	1	2	5369.0367	-0.7
6	3	4	5	4	1	5629.9578	-0.1
4	1	3	3	0	3	5643.9203	-3.1
6	2	5	5	3	2	5665.8513	0.9
4	2	2	3	1	2	5670.9181	-1.4
5	0	5	4	1	4	5788.1073	0.8
5	1	5	4	1	4	5802.0441	-3.4
5	0	5	4	0	4	5822.3639	-1.7
5	1	4	4	2	3	5833.8332	-5.2
5	1	5	4	0	4	5836.3063	-0.4
6	3	3	5	4	2	5912.1981	-2.9
4	2	3	3	1	3	5926.5817	1.2
5	2	4	4	2	3	6055.5887	0.0
4	3	2	3	2	1	6097.4719	0.9
4	3	1	3	2	1	6127.2211	-3.9
5	3	3	4	3	2	6162.1676	-0.2
5	4	2	4	4	1	6167.3389	1.7
5	4	1	4	4	0	6172.3370	3.7
5	1	4	4	1	3	6191.6687	3.7
4	3	2	3	2	2	6213.6677	0.2
4	2	2	3	1	3	6228.4593	-3.3
5	3	2	4	3	1	6240.6830	-3.3
4	3	1	3	2	2	6243.4197	-1.8
5	2	3	4	2	2	6339.5536	0.0
5	2	4	4	1	3	6413.4189	3.6
7	4	4	6	5	1	6437.3116	0.3
7	2	6	6	3	3	6492.1026	0.0
7	4	3	6	5	2	6527.4297	5.8
4	4	1	3	3	0	6668.9882	-0.8
4	4	0	3	3	0	6669.6309	-4.3
4	4	1	3	3	1	6673.4416	0.6
4	4	0	3	3	1	6674.0858	-1.2
6	2	4	5	3	3	6727.5605	-1.2
7	3	5	6	4	2	6787.9993	-2.0
6	0	6	5	1	5	6930.8389	0.0
6	1	6	5	1	5	6936.1237	1.5
6	0	6	5	0	5	6944.7815	1.6
6	1	6	5	0	5	6950.0664	3.1
5	2	3	4	1	3	6999.2577	-4.7
6	1	5	5	2	4	7107.0058	-2.6
8	5	4	7	6	1	7184.5319	0.8
5	3	3	4	2	2	7204.7849	-1.3
8	5	3	7	6	2	7206.0891	-3.1
6	2	5	5	2	4	7225.2043	1.6
5	3	2	4	2	2	7313.0586	-0.2
5	2	4	4	1	4	7320.2757	1.9
6	1	5	5	1	4	7328.7585	-0.3
4	3	2	3	0	3	7346.2501	-0.4
7	3	4	6	4	3	7366.9913	-4.7
6	3	4	5	3	3	7385.0291	-1.2

Table S10. Continued.

J'	K'_a	K'_c	J''	K''_a	K''_c	Obs.	Res.
6	5	2	5	5	1	7398.8822	0.7
6	5	1	5	5	0	7399.7084	2.5
6	4	3	5	4	2	7413.2840	4.9
6	4	2	5	4	1	7434.3500	0.6
6	2	5	5	1	4	7446.9525	-0.5
5	3	3	4	2	3	7506.6707	2.2
6	3	3	5	3	2	7553.3560	-2.6
6	2	4	5	2	3	7592.7900	-4.2
5	3	2	4	2	3	7614.9383	-2.7
8	4	5	7	5	2	7682.9721	-2.2
8	3	6	7	4	3	7848.1424	-10.2
5	4	2	4	3	1	7881.8398	-4.1
5	4	1	4	3	1	7887.4671	-19.1
5	2	3	4	1	4	7906.1172	-3.6
5	4	2	4	3	2	7911.5954	-2.6
5	4	1	4	3	2	7917.2407	0.4
8	4	4	7	5	3	7918.3897	3.7
7	0	7	6	1	6	8065.0525	2.3
7	1	7	6	1	6	8066.9634	3.1
7	0	7	6	0	6	8070.3369	3.4
7	1	7	6	0	6	8072.2464	2.7
7	2	5	6	3	4	8144.6967	0.0
14	1	14	13	2	11	8150.4642	-0.6
6	3	4	5	2	3	8250.2631	0.1
7	1	6	6	2	5	8324.0619	1.1
7	2	6	6	2	5	8379.6091	-1.6
5	5	1	4	4	0	8401.4692	-1.6
5	5	0	4	4	1	8402.1980	-3.6
7	1	6	6	1	5	8442.2559	0.9
7	2	6	6	1	5	8497.8105	5.5
7	3	5	6	3	4	8592.3887	-4.0
7	5	3	6	5	2	8647.1141	-1.9
7	5	2	6	5	1	8651.4564	0.8
7	4	4	6	4	3	8658.6020	-0.5
7	4	3	6	4	2	8721.0982	4.5
7	2	5	6	2	4	8802.1694	4.1
7	3	4	6	3	3	8868.0817	7.6
6	4	3	5	3	2	9054.4355	-1.1
6	4	2	5	3	2	9081.1489	-0.3
6	3	3	5	2	4	9112.7149	3.9
6	4	3	5	3	3	9162.7094	0.1
6	4	2	5	3	3	9189.4190	-2.7
8	0	8	7	1	7	9195.6289	-1.8
8	1	8	7	1	7	9196.2967	-1.9
8	0	8	7	0	7	9197.5413	0.4
8	1	8	7	0	7	9198.2082	-0.5
7	3	5	6	2	4	9249.8686	7.1
9	4	5	8	5	4	9391.2661	8.8
8	1	7	7	2	6	9498.5667	5.1
8	2	6	7	3	5	9512.9267	2.0
8	2	7	7	2	6	9522.4249	-1.3
8	1	7	7	1	6	9554.1110	-0.5
8	2	7	7	1	6	9577.9739	-2.2
6	5	2	5	4	1	9628.0284	9.3
6	5	1	5	4	2	9634.5755	5.2
8	3	6	7	3	5	9781.2421	-2.8
8	7	2	7	7	1	9859.8812	-0.4
8	7	1	7	7	0	9859.8812	-0.4
8	4	5	7	4	4	9897.1185	-0.2
8	5	4	7	5	3	9899.8736	12.1
8	5	3	7	5	2	9916.0457	3.9
10	5	5	9	6	4	9916.5141	-9.7
8	2	6	7	2	5	9960.6139	-6.8
8	4	4	7	4	3	10038.0854	7.3
6	6	1	5	5	0	10131.7417	-11.5
6	6	0	5	5	0	10131.7417	-11.5
6	6	1	5	5	1	10131.8456	-0.5
6	6	0	5	5	1	10131.8456	-0.5
8	3	5	7	3	4	10156.0433	11.8
7	4	4	6	3	3	10159.6936	13.0
8	3	6	7	2	5	10228.9399	-1.1
9	0	9	8	1	8	10324.7698	-0.9
9	1	9	8	1	8	10324.9970	-1.5
9	0	9	8	0	8	10325.4374	-1.2
9	1	9	8	0	8	10325.6653	-1.2
9	3	6	8	4	5	10366.7899	0.1
7	4	3	6	3	4	10525.4834	-1.5
9	1	8	8	2	7	10648.1380	-8.8
9	2	8	8	2	7	10657.7758	-5.7
9	1	8	8	1	7	10672.0114	0.0
9	2	8	8	1	7	10681.6438	-2.5
7	3	4	6	2	5	10755.6020	19.6
9	2	7	8	3	6	10809.6310	6.5
7	5	3	6	4	2	10840.7973	11.4
7	5	2	6	4	3	10872.7601	13.2
9	3	7	8	3	6	10951.4644	4.9
9	4	6	8	4	5	11122.4188	1.5
9	6	4	8	6	3	11129.4900	11.8
9	6	3	8	6	2	11132.8838	5.7
9	5	5	8	5	4	11154.1282	3.3

Table S10. Continued.

J'	K'_a	K'_c	J''	K''_a	K''_c	Obs.	Res.
8	4	5	7	3	4	11188.7256	0.4
9	3	7	8	2	6	11219.7800	0.2
7	6	2	6	5	1	11361.3975	-7.5
7	6	1	6	5	2	11362.4350	-11.4
9	4	5	8	4	4	11372.7399	6.9
9	3	6	8	3	5	11399.4988	15.3
10	0	10	9	1	9	11453.3470	-4.8
10	1	10	9	1	9	11453.4147	-13.3
10	0	10	9	0	9	11453.5706	-9.1
10	1	10	9	0	9	11453.6534	-2.5
10	1	9	9	2	8	11785.3074	3.8
10	1	9	9	1	8	11794.9371	-1.2
10	2	9	9	1	8	11798.6421	-16.0
10	3	7	9	4	6	11832.7186	1.9
7	7	1	6	6	0	11861.4565	3.6
7	7	1	6	6	1	11861.4565	3.6
7	7	0	6	6	0	11861.4565	3.6
7	7	0	6	6	1	11861.4565	3.6
8	5	3	8	4	4	2002.9018	-2.5
7	1	6	7	0	7	2066.3123	-4.9
8	3	6	8	2	7	2082.5048	-3.3
7	2	6	7	1	7	2119.9498	-7.3
7	5	2	7	4	3	2124.9461	5.4
9	4	6	9	3	7	2130.7268	6.6
6	5	1	6	4	2	2194.5809	2.1
7	5	3	7	4	4	2208.8965	0.6
8	5	4	8	4	5	2211.6412	2.6
6	5	2	6	4	3	2220.3837	1.4
5	5	0	5	4	1	2229.2224	0.2
5	5	1	5	4	2	2234.7774	-2.3
9	5	5	9	4	6	2243.3453	-0.8
9	2	7	9	1	8	2243.9877	1.8
10	5	6	10	4	7	2318.4220	4.1
10	4	7	10	3	8	2354.8005	10.6
9	3	7	9	2	8	2376.1885	2.5
8	1	7	8	0	8	2422.8857	-2.2
8	2	7	8	1	8	2446.0825	-2.2
9	6	3	9	5	4	2603.1976	-0.1
8	6	2	8	5	3	2671.5040	-2.5
8	6	3	8	5	4	2692.0317	-4.6
10	3	8	10	2	9	2692.7586	6.6
7	6	1	7	5	2	2710.0846	2.6
7	6	2	7	5	3	2715.1971	-0.8
6	6	0	6	5	1	2732.0619	5.1
6	6	1	6	5	2	2732.9499	-5.3
9	1	8	9	0	9	2769.4537	-7.1
9	2	8	9	1	9	2778.8632	-4.5
11	7	4	11	6	5	3079.6483	-3.0
16	8	8	16	7	9	3086.8158	10.4
10	1	9	10	0	10	3110.8156	-3.8
11	7	5	11	6	6	3126.6091	2.2
10	7	3	10	6	4	3146.0608	9.0
10	7	4	10	6	5	3161.4338	3.3
13	3	10	13	2	11	3180.1907	-19.4
9	7	2	9	6	3	3187.5853	-1.6
9	7	3	9	6	4	3191.7457	0.3
8	7	2	8	6	3	3215.2656	-6.3
7	7	0	7	6	1	3231.9655	-1.9
7	7	1	7	6	2	3232.0917	-7.0
11	1	10	11	0	11	3449.6384	-8.7
15	8	8	15	7	9	3502.7782	-20.5
14	3	11	14	2	12	3552.0980	-4.5
13	8	5	13	7	6	3553.7563	-6.7
12	8	4	12	7	5	3618.5423	-3.1
12	8	5	12	7	6	3629.6271	0.6
11	8	3	11	7	4	3662.5215	1.1
11	8	4	11	7	5	3665.6819	4.1
10	8	2	10	7	3	3693.3707	-9.3
10	8	3	10	7	4	3694.1370	11.0
13	3	11	13	2	12	3696.4313	9.7
9	8	1	9	7	2	3715.3557	4.6
9	8	2	9	7	3	3715.4828	-6.1
8	8	0	8	7	1	3730.8853	-1.8
8	8	1	8	7	2	3730.8853	-1.8
9	9	0	9	8	1	4229.1630	3.1
9	9	1	9	8	2	4229.1630	3.1
12	11	1	12	10	2	5211.5108	-7.4
12	11	2	12	10	3	5211.5108	-7.4
11	11	0	11	10	1	5224.0051	11.2
11	11	1	11	10	2	5224.0051	11.2

Table S11. Observed frequencies (MHz) and residuals (kHz) for the $^{34}S_1$ isotopologue of WS_4-I for $J'K_a'K_c' \leftarrow J''K_a''K_c''$ transitions.

J'	K_a'	K_c'	J''	K_a''	K_c''	Obs.	Res.
2	1	2	1	0	1	2537.3048	-0.1
3	0	3	2	1	2	3422.3986	-0.4
3	1	3	2	0	2	3628.5756	-3.9
3	2	2	2	1	1	4236.4017	4.2
4	1	3	3	2	2	4491.4059	9.0
4	0	4	3	1	3	4606.8942	-1.4
4	0	4	3	0	3	4679.5905	-5.3
4	1	4	3	0	3	4712.9549	-4.8
4	2	3	3	2	2	4836.1991	0.3
3	3	1	2	2	0	4844.1424	0.7
3	3	0	2	2	1	4871.9479	-0.9
4	1	3	3	1	2	4971.0761	3.5
4	2	2	3	2	1	5010.9190	1.9
5	2	3	4	3	2	5284.9597	-19.9
4	2	3	3	1	2	5315.8755	1.0
5	0	5	4	1	4	5762.7852	0.0
5	0	5	4	0	4	5796.1455	-3.6
5	1	4	4	2	3	5801.3325	5.7
5	1	5	4	0	4	5809.8150	-5.4
4	3	2	3	2	1	6010.0319	-13.2
5	1	4	4	1	3	6146.1345	5.8
4	3	1	3	2	2	6146.6936	2.0
5	2	3	4	2	2	6284.1101	2.4
5	2	4	4	1	3	6361.1043	-2.4
4	4	1	3	3	0	6554.8377	0.0
4	4	0	3	3	1	6559.5574	-3.0
6	0	6	5	1	5	6901.6414	0.0
6	1	6	5	1	5	6906.8584	0.2
6	0	6	5	0	5	6915.3197	7.0
6	1	6	5	0	5	6920.5310	1.5
6	1	5	5	2	4	7065.1730	8.9
5	3	3	4	2	2	7115.5429	-5.5
6	2	5	5	2	4	7180.5919	0.5
6	1	5	5	1	4	7280.1400	-2.1
6	3	4	5	3	3	7330.9505	0.2
6	2	5	5	1	4	7395.5655	-3.9
6	3	3	5	3	2	7488.2646	3.1
5	3	2	4	2	3	7500.0103	4.5
6	2	4	5	2	3	7527.9960	-0.5
5	4	2	4	3	1	7759.8697	-0.9
5	4	1	4	3	2	7792.6788	-1.2
7	0	7	6	1	6	8032.2472	-1.0
7	1	7	6	0	6	8039.3676	3.8
6	3	4	5	2	3	8162.4031	12.0
5	5	1	4	4	0	8255.7903	0.2
5	5	0	4	4	1	8256.4614	1.0
7	2	6	6	1	5	8445.9258	3.5
6	4	2	5	3	3	9052.6626	-1.8
8	0	8	7	1	7	9159.3155	4.3
8	1	8	7	0	7	9161.8760	-2.4
6	5	2	5	4	1	9473.5628	0.6
6	5	1	5	4	2	9479.5737	0.0
6	6	1	5	5	0	9954.6878	-3.1
6	6	0	5	5	1	9954.7768	-0.4
9	1	9	8	0	8	10285.8529	0.2
9	2	8	8	1	7	10624.7025	-7.8
7	7	1	6	6	0	11653.0495	1.7
7	7	0	6	6	1	11653.0495	1.7

Table S12. Observed frequencies (MHz) and residuals (kHz) for the $^{34}S_2$ isotopologue of WS_4-I for $J'K_a'K_c' \leftarrow J''K_a''K_c''$ transitions.

J'	K_a'	K_c'	J''	K_a''	K_c''	Obs.	Res.
2	2	1	1	1	0	3145.0919	-0.9
3	0	3	2	1	2	3361.3743	0.2
3	0	3	2	0	2	3513.9930	0.2
3	2	2	2	2	1	3594.8961	3.3
3	1	3	2	0	2	3601.0989	1.2
3	2	1	2	2	0	3675.7586	-1.1
3	1	2	2	1	1	3712.7276	-7.2
3	2	2	2	1	1	4254.1074	-0.1
4	1	3	3	2	2	4374.1931	2.4
4	0	4	3	1	3	4536.7731	-2.0
4	0	4	3	0	3	4623.8793	-0.7
4	1	4	3	0	3	4666.0463	-0.9
4	2	3	3	2	2	4775.6345	-5.0
3	3	1	2	2	0	4907.0403	1.9
4	2	3	3	1	2	5317.0120	-0.2
5	1	4	4	2	3	5680.3812	-1.7
5	0	5	4	1	4	5682.1097	-4.3
5	1	5	4	1	4	5700.3519	2.5
5	0	5	4	0	4	5724.2953	14.0
5	1	5	4	0	4	5742.5158	-0.8
5	2	4	4	2	3	5942.3692	-4.7
4	3	2	3	2	1	6060.0926	1.1
5	1	4	4	1	3	6081.8355	3.7
4	3	1	3	2	2	6187.1159	3.2
5	2	3	4	2	2	6205.6801	-2.7
5	2	4	4	1	3	6343.8238	0.9
6	2	4	5	3	3	6493.1643	-2.2
4	4	1	3	3	0	6645.0086	-0.5
4	4	0	3	3	1	6649.0457	-0.2
6	0	6	5	1	5	6808.2518	-2.6
6	1	6	5	1	5	6815.5908	-1.2
6	1	6	5	0	5	6833.8266	-0.8
6	1	5	5	2	4	6945.0410	1.3
7	3	4	6	4	3	7059.3595	-1.4
6	2	5	5	2	4	7093.8394	-3.1
5	3	3	4	2	2	7153.5638	1.3
6	1	5	5	1	4	7207.0317	1.0
6	2	5	5	1	4	7355.8344	0.7
6	3	3	5	3	2	7382.3268	-0.2
6	2	4	5	2	3	7441.0467	0.4
5	3	2	4	2	3	7514.0634	3.5
5	2	3	4	1	4	7747.6031	4.2
5	4	2	4	3	1	7836.1904	0.7
5	4	1	4	3	2	7864.3417	-3.4
7	2	5	6	3	4	7893.2096	1.0
7	0	7	6	1	6	7924.5142	1.8
7	1	7	6	1	6	7927.3246	-2.5
7	0	7	6	0	6	7931.8555	5.5
7	1	7	6	0	6	7934.6644	-0.3
7	1	6	6	2	5	8156.1931	2.0
5	5	1	4	4	0	8373.6117	1.5
5	5	0	4	4	1	8374.1384	-1.2
6	4	3	5	3	2	8993.7841	-7.8
8	0	8	7	1	7	9036.2795	0.7
8	1	8	7	0	7	9040.1417	4.4
6	4	2	5	3	3	9102.3712	-1.0
8	2	7	7	1	6	9431.1406	0.4
6	5	2	5	4	1	9576.0536	6.9
6	5	1	5	4	2	9580.8020	-5.2
6	6	1	5	5	0	10100.3657	-4.6
6	6	0	5	5	1	10100.4277	-5.6
9	0	9	8	1	8	10146.1531	0.9
9	1	9	8	0	8	10147.5672	-6.5
7	5	2	6	4	3	10791.2491	4.2
7	6	2	6	5	1	11305.1823	-2.0
7	7	1	6	6	0	11826.6311	9.8
7	7	0	6	6	1	11826.6311	2.8

Table S13. Observed frequencies (MHz) and residuals (kHz) for the $^{34}S_3$ isotopologue of WS_4-I for $J'K'_aK'_c \leftarrow J''K''_aK''_c$ transitions.

J'	K'_a	K'_c	J''	K''_a	K''_c	Obs.	Res.
2	1	2	1	0	1	2514.3324	0.8
3	0	3	2	1	2	3414.1282	0.1
3	1	3	2	0	2	3589.7717	0.3
3	2	2	2	1	1	4206.2216	0.0
4	0	4	3	1	3	4580.5467	-0.7
4	1	4	3	1	3	4604.2238	-1.6
4	0	4	3	0	3	4638.4065	3.1
3	2	1	2	1	2	4653.0197	-8.3
4	1	4	3	0	3	4662.0793	-2.0
3	3	1	2	2	0	4822.5984	0.8
3	3	0	2	2	1	4860.5037	0.0
4	1	3	3	1	2	4975.2968	0.3
4	2	2	3	2	1	5047.3260	-1.5
4	2	3	3	1	2	5266.3295	5.1
5	0	5	4	1	4	5717.7972	1.0
5	0	5	4	0	4	5741.4706	-3.4
5	1	5	4	0	4	5750.1286	0.0
5	1	4	4	2	3	5837.1011	0.7
4	3	2	3	2	1	5974.7492	0.4
5	2	4	4	2	3	6000.5278	0.5
5	1	4	4	1	3	6128.1277	-0.3
4	3	1	3	2	2	6158.6213	0.0
5	3	2	4	3	1	6235.0078	-1.6
5	2	4	4	1	3	6291.5538	-1.3
5	2	3	4	2	2	6320.5733	4.1
4	4	1	3	3	0	6529.6334	1.9
4	4	0	3	3	1	6537.1634	6.5
6	0	6	5	1	5	6839.9476	-0.6
6	1	6	5	1	5	6842.8988	-0.2
6	0	6	5	0	5	6848.5986	-4.2
6	1	6	5	0	5	6851.5526	-1.0
5	3	3	4	2	2	7055.6115	-1.8
6	1	5	5	2	4	7073.0793	-2.5
6	1	5	5	1	4	7236.5111	2.1
6	2	5	5	1	4	7314.1200	0.4
6	3	4	5	3	3	7337.7422	11.0
6	2	4	5	2	3	7551.5855	-15.5
6	3	3	5	3	2	7552.8468	1.9
5	3	2	4	2	3	7562.7333	0.8
5	4	2	4	3	1	7728.8477	-0.2
5	4	1	4	3	2	7780.5565	-0.7
7	0	7	6	1	6	7955.6577	2.4
7	1	7	6	1	6	7956.6171	1.0
7	0	7	6	0	6	7958.6063	0.1
7	1	7	6	0	6	7959.5682	1.3
6	3	4	5	2	3	8072.7787	3.3
7	2	5	6	3	4	8205.4903	-0.3
5	5	1	4	4	0	8224.5464	-6.0
5	5	0	4	4	1	8225.8049	3.2
7	2	6	6	1	5	8362.2278	8.7
6	4	3	5	3	2	8874.5337	-0.7
7	3	5	6	2	4	9048.2776	-8.1
6	4	2	5	3	3	9067.1514	4.4
8	0	8	7	1	7	9068.9135	5.7
8	1	8	7	0	7	9070.1618	-9.5
8	1	7	7	2	6	9394.9409	2.8
6	5	2	5	4	1	9442.5761	4.5
6	5	1	5	4	2	9453.6924	-2.5
6	6	1	5	5	0	9916.5164	0.0
6	6	0	5	5	1	9916.7011	-3.4
7	4	4	6	3	3	9940.1194	-3.1
7	7	1	6	6	0	11607.7167	-5.2
7	7	0	6	6	1	11607.7554	6.7

Table S14. Observed frequencies (MHz) and residuals (kHz) for the $^{34}S_4$ isotopologue of WS_4-I for $J'K_a'K_c' \leftarrow J''K_a''K_c''$ transitions.

J'	K_a'	K_c'	J''	K_a''	K_c''	Obs.	Res.
2	2	1	1	1	0	3143.9823	3.7
3	0	3	2	1	2	3360.9142	-1.2
3	1	3	2	1	2	3448.0624	-0.3
3	1	3	2	0	2	3600.6525	1.6
3	1	2	2	1	1	3711.7747	0.3
3	2	2	2	1	1	4252.9349	0.1
4	1	3	3	2	2	4373.2482	3.9
4	0	4	3	1	3	4536.2205	-0.7
4	0	4	3	0	3	4623.3671	-1.5
4	1	4	3	0	3	4665.5948	4.8
4	2	3	3	2	2	4774.7095	-6.4
3	3	1	2	2	0	4905.1366	-2.7
4	1	3	3	1	2	4914.4094	4.8
3	3	0	2	2	1	4930.7670	2.5
4	2	3	3	1	2	5315.8755	-0.7
5	1	4	4	2	3	5679.1257	0.0
5	0	5	4	1	4	5681.5102	-1.5
5	0	5	4	0	4	5723.7442	10.9
5	1	5	4	0	4	5742.0067	0.0
5	2	4	4	2	3	5941.3030	-2.3
5	3	3	4	3	2	6036.4907	-5.9
4	3	2	3	2	1	6058.0876	1.0
5	1	4	4	1	3	6080.5971	-0.2
4	3	1	3	2	2	6184.6888	2.7
5	2	3	4	2	2	6203.9593	0.9
5	2	4	4	1	3	6342.7779	0.9
6	2	4	5	3	3	6491.4585	3.3
4	4	1	3	3	0	6642.3539	2.7
4	4	0	3	3	1	6646.3655	-3.9
6	0	6	5	1	5	6807.6127	-0.9
6	1	6	5	1	5	6814.9711	-1.3
6	0	6	5	0	5	6825.8943	7.0
6	1	6	5	0	5	6833.2436	-2.4
6	1	5	5	2	4	6943.6408	2.9
5	3	3	4	2	2	7151.5980	0.3
6	1	5	5	1	4	7205.8166	-0.9
6	3	4	5	3	3	7237.2827	0.9
6	3	3	5	3	2	7380.2204	-7.2
6	2	4	5	2	3	7439.1002	5.7
5	3	2	4	2	3	7510.9466	3.1
5	4	2	4	3	1	7833.3259	0.9
5	4	1	4	3	2	7861.3523	-0.8
7	2	5	6	3	4	7891.0266	-0.1
7	0	7	6	1	6	7923.8276	-1.6
7	1	7	6	1	6	7926.6500	-4.3
7	0	7	6	0	6	7931.1826	-5.4
7	1	7	6	0	6	7934.0107	-2.5
7	1	6	6	2	5	8154.7727	4.2
6	3	4	5	2	3	8184.9279	6.8
5	5	1	4	4	0	8370.2158	-2.6
5	5	0	4	4	1	8370.7414	-3.5
7	2	6	6	1	5	8378.7228	3.1
7	3	5	6	3	4	8425.0608	-12.4
8	0	8	7	1	7	9035.5376	-10.5
6	4	2	5	3	3	9098.9586	2.6
6	5	1	5	4	2	9577.1353	-0.3
6	6	1	5	5	0	10096.2388	-11.8
6	6	0	5	5	1	10096.3140	0.8
9	0	9	8	1	8	10145.3792	8.0
9	1	9	8	0	8	10146.8017	2.4
7	7	1	6	6	0	11821.7845	7.9
7	7	0	6	6	1	11821.7845	7.9

Table S15. Observed frequencies (MHz) and residuals (kHz) for the parent species of W4S-I for $J'K'_aK'_c \leftarrow J''K''_aK''_c$ transitions.

J'	K'_a	K'_c	J''	K''_a	K''_c	Obs.	Res.
1	0	1	0	0	0	2633.1227	-3.0
2	1	2	1	1	1	5243.9573	0.4
2	0	2	1	0	1	5265.4227	4.5
2	1	1	1	1	0	5288.0961	9.4
3	1	3	2	1	2	7865.3565	-6.1
3	0	3	2	0	2	7896.0458	-2.8
3	2	2	2	2	1	7898.0742	0.9
3	2	1	2	2	0	7901.2421	-11.2
3	1	2	2	1	1	7931.5447	4.9
4	1	4	3	1	3	10486.1131	8.7
4	0	4	3	0	3	10524.2159	-2.0
4	2	3	3	2	2	10529.9992	-3.2
4	3	2	3	3	1	10530.1887	11.0
4	3	1	3	3	0	10530.2763	-8.0
4	2	2	3	2	1	10537.9178	2.0
4	1	3	3	1	2	10574.2532	-4.2

Table S16. Theoretical coordinates for WS_4-I at the ωB97X-V/def2-TZVP level of theory.

Atom	x (a.u.)	y (a.u.)	z (a.u.)
O	0.130225	-1.930526	-1.485629
S	-2.781660	-0.089617	-0.863957
H	-2.674938	-0.584248	0.377161
H	-1.733371	-0.807621	-1.305056
S	-0.061498	-1.322823	1.931269
S	2.790491	-0.038162	-0.727029
S	-0.025523	2.590372	0.430843
H	0.954121	-1.415006	-1.481886
H	0.266518	-2.646782	-2.112779
H	-0.041493	-2.495896	2.575730
H	-0.084663	-1.892191	0.713541
H	-0.002298	1.933022	1.598202
H	-1.010565	1.816631	-0.058892
H	1.924453	0.960978	-0.475589
H	2.586178	-0.590902	0.477996

Table S17. Theoretical coordinates for WS_4-II at the ωB97X-V/def2-TZVP level of theory.

Atom	x (a.u.)	y (a.u.)	z (a.u.)
O	0.397878	-1.224972	-1.803993
S	-0.773975	1.578338	1.644386
H	0.512885	1.439871	1.284774
H	-0.953382	0.248156	1.700495
S	-0.576962	-2.504141	1.267878
S	2.792438	0.038969	0.419773
S	-1.530380	1.494538	-2.277707
H	-0.172117	-0.484217	-2.070649
H	0.580635	-1.722813	-2.606663
H	-0.292841	-2.153342	0.000108
H	-1.855194	-2.785953	0.987475
H	-1.432859	1.598337	-0.937942
H	-0.501762	2.328210	-2.479994
H	1.972346	-0.514108	-0.492147
H	2.445514	-0.839176	1.370009

Table S18. Theoretical coordinates for WS_4-III at the ωB97X-V/def2-TZVP level of theory.

Atom	x (a.u.)	y (a.u.)	z (a.u.)
S	-0.555264	-1.113702	-1.834313
H	-0.396897	-0.754852	-0.544974
H	-0.173038	0.086419	-2.290775
O	-0.306092	-0.014300	1.462082
H	-1.252365	0.168055	1.548735
H	0.105686	0.838644	1.261278
S	1.666841	2.164374	-0.229269
H	2.410230	2.875036	0.627668
H	2.371604	1.036019	-0.030359
S	-3.587930	0.478740	0.506130
H	-4.063633	-0.718792	0.869654
H	-2.997337	-0.012573	-0.594616
S	2.742824	-1.587936	0.686494
H	2.074216	-1.877306	-0.438846
H	1.612213	-1.340281	1.367619

Table S19. Theoretical coordinates for W_4S-I at the ωB97X-V/def2-TZVP level of theory.

Atom	x (a.u.)	y (a.u.)	z (a.u.)
O	1.046537	1.930910	0.531539
S	-2.176272	0.001708	0.000644
H	-1.252453	0.251105	-0.939455
H	-1.252396	-0.248591	0.940437
O	1.042025	-1.932188	-0.532742
O	0.951011	-0.517772	1.787620
H	1.505173	-0.849529	2.498889
H	1.045596	-1.143230	1.032332
O	0.949503	0.516568	-1.787809
H	1.503941	0.846326	-2.499812
H	1.048717	1.140797	-1.032065
H	1.067246	-1.145854	-1.117867
H	0.200235	-2.361288	-0.718380
H	1.070791	1.144435	1.116412
H	0.206355	2.362457	0.718682

Table S20. Theoretical coordinates for W_4S-II at the ωB97X-V/def2-TZVP level of theory.

Atom	x (a.u.)	y (a.u.)	z (a.u.)
O	0.785097	0.293691	-1.510249
S	-2.521502	1.093368	-0.780341
H	-1.619847	1.282823	0.198587
H	-2.863455	-0.120632	-0.329990
O	1.817928	-1.983059	-0.417424
O	1.563910	-0.877733	2.064500
H	1.725409	-1.374762	1.230151
H	1.085190	-1.475493	2.644800
O	0.490876	1.403127	1.050970
H	0.936247	2.174261	1.412684
H	0.847385	0.623455	1.536791
H	1.445885	-1.232895	-0.935148
H	2.677188	-2.173565	-0.803329
H	0.752081	0.826106	-0.691596
H	-0.126746	0.297607	-1.827022

Table S21. Theoretical coordinates for W_4S-III at the ωB97X-V/def2-TZVP level of theory.

Atom	x (a.u.)	y (a.u.)	z (a.u.)
O	0.450132	0.773647	1.784212
S	-2.266878	0.005382	-0.185948
H	-1.887887	-1.154445	0.369502
H	-1.204204	0.009419	-1.007544
O	0.656096	-1.832999	0.752028
O	1.033893	-0.482984	-1.612344
H	1.342085	-0.629089	-2.509852
H	1.427757	0.364552	-1.300121
O	1.800832	1.774035	-0.357223
H	2.675449	2.099898	-0.130554
H	1.363384	1.527503	0.491669
H	1.287125	-2.519969	0.981827
H	0.872802	-1.540934	-0.156498
H	-0.480351	0.932125	1.575097
H	0.566043	-0.180362	1.607779
